# Mapping Pathological Phenotypes in a Mouse Model of CDKL5 Disorder

**DOI:** 10.1371/journal.pone.0091613

**Published:** 2014-05-16

**Authors:** Elena Amendola, Yang Zhan, Camilla Mattucci, Enrico Castroflorio, Eleonora Calcagno, Claudia Fuchs, Giuseppina Lonetti, Davide Silingardi, Alexei L. Vyssotski, Dominika Farley, Elisabetta Ciani, Tommaso Pizzorusso, Maurizio Giustetto, Cornelius T. Gross

**Affiliations:** 1 Mouse Biology Unit, European Molecular Biology Laboratory (EMBL), Monterotondo, Italy; 2 Department of Neuroscience and National Institute of Neuroscience, University of Turin, Turin, Italy; 3 Department of Biomedical and Neuromotor Sciences, University of Bologna, Bologna, Italy; 4 Institute of Neuroscience, National Research Council (CNR), Pisa, Italy; 5 Department of Neuroscience, Psychology, Drug Research and Child Health NEUROFARBA University of Florence, Florence, Italy; 6 Institute of Neuroinformatics, University of Zürich and Swiss Federal Institute of Technology (ETH), Zurich, Switzerland; Institute of Genetics and Biophysics, Italy

## Abstract

Mutations in cyclin-dependent kinase-like 5 (*CDKL5*) cause early-onset epileptic encephalopathy, a neurodevelopmental disorder with similarities to Rett Syndrome. Here we describe the physiological, molecular, and behavioral phenotyping of a *Cdkl5* conditional knockout mouse model of CDKL5 disorder. Behavioral analysis of constitutive *Cdkl5* knockout mice revealed key features of the human disorder, including limb clasping, hypoactivity, and abnormal eye tracking. Anatomical, physiological, and molecular analysis of the knockout uncovered potential pathological substrates of the disorder, including reduced dendritic arborization of cortical neurons, abnormal electroencephalograph (EEG) responses to convulsant treatment, decreased visual evoked responses (VEPs), and alterations in the Akt/rpS6 signaling pathway. Selective knockout of *Cdkl5* in excitatory and inhibitory forebrain neurons allowed us to map the behavioral features of the disorder to separable cell-types. These findings identify physiological and molecular deficits in specific forebrain neuron populations as possible pathological substrates in CDKL5 disorder.

## Introduction

Mutations in the X-linked cyclin-dependent kinase-like 5 (*CDKL5*) gene cause early-onset epileptic encephalopathy [1]. Although CDKL5 disorder shares several features with Rett Syndrome, a neurodevelopmental disorder caused by mutations in the X-linked *MECP2* gene [2], recent work assessing data from 86 subjects has argued that it should be considered a distinct clinical entity, primarily due to its early onset and lack of clinical regression following a period of normal development [3]. The primary clinical features of CDKL5 disorder are seizures initiating in the first few months of life, stereotypical hand movements, motor rigidity, and deficient language acquisition [3], [4]. Several additional features have been noted in some carriers, including gastrointestinal problems, bruxism [4], [5], and a characteristic sideways glance [4]. The disorder is most frequently associated with nonsense or putative detrimental missense mutations and is thought to be caused by a loss of CDKL5 function, although no clear relationship between the type or location of mutations and symptom severity has been reported [6]. The disorder is more frequently reported in females (8∶1) [3], probably due to the more severe consequences of dominant X-linked mutations in males than in females. CDKL5 mRNA is expressed in brain, testes, and thymus [7], [8] and the protein product is found in both the cytoplasm and nucleus, where it colocalizes with nuclear speckles [9], [10]. The mouse *Cdkl5* gene expresses two isoforms, with expression segregated to neurons and astrocytes [11]. Data demonstrate that CDKL5 can bind to and phosphorylate MECP2 *in vitro*, suggesting a possible molecular link between CDKL5 disorder and Rett Syndrome [8].

Here we generated and characterized mice carrying a targeted conditional knockout allele of *Cdkl5*. Behavioral characterization was carried out to identify features that mimic the clinical features described in CDKL5 disorder, including seizures, motor behavior, and eye tracking. Several physiological substrates were examined, including spontaneous and convulsant-induced electroencephalograph (EEG) activity and visual evoked potentials (VEPs). Anatomical analysis was aimed at identifying aberrant morphological features of neurons, including cortical neuron dendritic arborization, reported following the developmental knockdown of Cdkl5 in rat [11], in *Mecp2* knockout mice [12]–[14], and in postmortem samples from MECP2 carriers [15]. Molecular analyses were carried out on several signaling pathways identified to be altered in *Mecp2* knockout mice and thought to be relevant to Rett Syndrome. Finally, we used a conditional knockout approach to map the behavioral features identified to distinct populations of forebrain neurons. Our findings disclose a series of behavioral phenotypes homologous to those described in CDKL5 disorder and demonstrate the underlying neuronal cell-types and brain regions. In addition, our data reveal common deficits in a specific signaling pathway in *Cdkl5* and *Mecp2* knockout mouse models, suggesting potentially overlapping molecular deficits in CDKL5 disorder and Rett Syndrome.

## Methods

### Ethics statements

All procedures were approved by The Institutional Animal Care and Use Committee (IACUC) of The European Molecular Biology Laboratory (EMBL) and were conducted according to the Italian Ministry of Health and commensurate with NIH guidelines for the ethical treatment of animals (NIH publication No. 85-23, revised 2011). All surgery was performed under anesthesia with tribromoethanol 250 mg/Kg (avertin). After anesthesia, for fresh brains collections mice were sacrificed by cervical dislocation while for fixed tissues collections mice were perfused transcardially with 4% paraformaldehyde. All efforts were made to minimize suffering.

### Mouse strains and husbandry

All mice were handled according to protocols approved by the Italian Ministry of Health and commensurate with NIH guidelines for the ethical treatment of animals. Mice for testing were produced by crossing *Cdkl5* ^KO^/+ females with *Cdkl5* ^KO^/Y males and *Cdkl5* ^KO^/X females with +/Y male. Littermate controls were used for all experiments. A portion of the behavioral data derived from knockout mice containing the neomycin selection cassette. No difference in behavior was noted between this allele and the neo-negative allele and the results were combined. For dendritic reconstructions mice with sparse fluorescent labeling of cortical neurons were obtained by crossing *Thy1*::*GFP/Thy1::GFP*;+/Y males with +/+;*Cdkl5* ^KO^/X females and *Thy1*::*GFP/+*;*Cdkl5* ^KO^/Y males with +/+;*Cdkl5* ^KO^/X females. After weaning, mice were housed three to five per cage on a 12 h light/dark cycle (lights off at 19:00 h) in a temperature-controlled environment (21±2 C) with food and water provided *ad libitum*. For tissues collections all surgery was performed under anesthesia with tribromoethanol 250 mg/Kg (avertin). All efforts were made to minimize suffering.

### Generation of *Cdkl5* knockout mice

A 10 kb genomic fragment containing exon 4 of *Cdkl5* (ENSMUSE00000346596) was subcloned into a pDTA targeting plasmid by recombineering-mediated transfer from a 178-kb genomic fragment containing the C57BL/6J mouse *Cdkl5* locus (RP23-213O8, ChoriBACPAC, Oklahoma, CA). A loxP site was inserted 806 bp upstream of the exon by recombineering-mediated insertion of a loxP-flanked pEM7::kanamycin gene and subsequent Cre recombination. An FRT-flanked pEM7/PGK::neomycin selection cassette was inserted 347 bp downstream of exon 4. The plasmid was linearized with NruI before electroporation into ES cells (129/Sv×C57BL/6N, clone A8, gift of A. Wutz, Wellcome Trust Centre for Stem Cell Research, Stem Cell Institute, University of Cambridge). G418-resistent clones were identified and screened by long-range PCR. Hybridization with a specific probe for the 5′ and 3′ arms was used to confirm PCR results. Two independent positive ES cell clones were injected into C57BL/6N host embryos using a piezo-drill assisted 8-cell stage injection procedure developed at EMBL. Four out of five offspring (all >95% ES cell derived) provided germline transmission. Positive offspring were crossed to C57BL/6J congenic FLP-deleter mice [16] to remove the neomycin selection cassette and further crossed to C57BL/6J congenic Cre-deleter mice [17] to generate the *Cdkl5* null allele.

### Immunofluorescence, dendritic reconstruction

Mice were anesthetized (Avertin, Sigma-Aldrich, St. Louis, MO) and perfused transcardially with 4% paraformaldehyde at 2 months of life. Brains were removed from the skull and post-fixed overnight at 4 C. Coronal sections (60 and 250 µm for immunofluorescence and neuronal reconstruction, respectively) were cut on a vibratome (Leica Microsystems, Mannheim, Germany) in 0.1 M phosphate buffer. For immunofluorescence brain sections were washed in phosphate-buffered saline 0.5% Triton-X and transferred in a 15 mM sodium citrate solution, pH 8.0 for 30 min at 80 C, then washed in phosphate-buffered saline with 0.5% Triton-X and blocked with blocking buffer (2% BSA in phosphate-buffered saline, 100 mm glycine, 1% Triton-X), incubated with primary antibodies overnight at 4 C (rabbit anti-Cdkl5, 1∶250, Sigma-Aldrich; mouse anti-SC35, 1∶20, rabbit anti-MeCP2, 1∶1000, Cell Signaling, Danvers, MA), incubated with an appropriate Alexa Fluor secondary antibody (1 h at room temperature), stained with DAPI, and mounted in Moviol (Calbiochem, Nottingham, UK). Images were acquired on a confocal microscope (TCS SP5 AOBS, Leica Microsystems). For neuronal reconstruction, brain sections were washed in phosphate-buffered saline, stained with DAPI, and mounted in Moviol (Calbiochem). Total dendritic length and Sholl analysis was measured using ImageJ software from confocal images.

### Anatomical measurements

Mice were anesthetized (Avertin, Sigma-Aldrich) and perfused transcardially with 4% paraformaldehyde at 2 months of life. Brains were removed from the skull and post-fixed overnight at 4°C. The right hemisphere was dehydrated through a series of ascending ethanol concentrations, embedded in paraffin, and cut with a microtome (8 µm) and mounted on poly-lysine slides. One of 20 sections from the dentate gyrus was stained with toluidine blue according to the Nissl method. Bright field images (Leitz Diaplan, Wetzlar, Germany) were acquired with a Coolsnap-Pro digital camera (Media Cybernetics, Silver Spring, MD) and anatomical measurements carried out with Image Pro Plus software (Media Cybernetics).

### Immunohistochemistry

Animals were anesthetized with chloral hydrate and transcardially perfused with ice cold 4% paraformaldehyde in 0.1 M phosphate buffer (PB, pH 7.4). After perfusion, the brains were dissected and kept in the same fixative solution overnight at 4 C. After several washes in 0.1 M PB, brains were cryoprotected by immersion in 10%, 20%, and 30% sucrose solutions. One brain hemisphere was cut in 30 µm sections with a cryostat, collected in phosphate buffered saline and processed for free-floating immunohistochemistry as described [18]. After a blocking step in PBS, 10% NGS, 0.05% Triton X-100 sections were incubated overnight at room temperature with the following primary antibodies: rabbit anti-phospho-rpS6 (235/236) XP (1∶200); rabbit anti-phospho-rpS6 (240/244) XP (1∶800); rabbit anti-rpS6 (1∶100; Cell Signaling Technology) diluted in PBS, 3% NGS, 0.05% Triton X-100. Sections were washed in PBS, incubated for 1 hour with goat anti-rabbit biotinylated secondary antibodies (1∶250; Vector Labs, Burlingame, CA) and transferred to a solution containing a biotin-avidin complex (1∶100,Vector Labs). The peroxidase reaction product was visualized by incubation in a solution containing 3,3′-diaminobenzidine (0.05% DAB in Tris-HCI, pH 7.6) with 0.01% H_2_O_2_ for 3 min. Sections were mounted on gelatin-coated glass slides and observed with a light microscope (Eclipse 800, Nikon, Tokyo, Japan) equipped with a CCD camera (Axiocam HRc, Zeiss, Jena, Germany). Quantitation of immunolabeling experiments was carried out from optical density (OD) measurements on 10× micrographs using ImageJ by an operator blind to genotype. Measurements of OD in cortex were obtained from 100×50 µm measuring boxes that were randomly placed in each cortical layer. Histograms illustrate the average OD obtained from three repeated measures in 4–5 sections per experimental animal. The mean OD of the corpus callosum was subtracted as background staining.

### Western blotting

Mice at 2 months of life were decapitated and brains rapidly collected on ice and frozen in liquid nitrogen. One hemisphere was homogenized in lysis buffer (50 mM HEPES pH 7.0, 250 mM NaCl, 0.5% NP-40, 5 mM EDTA, 1 mM DTT) using an automated dounce. A protease/phosphatase inhibitor mix composed of 0.5 mM Na_3_VO_4_, 0.5 mM PMSF, protease inhibitor mixture (Roche Applied Sciences, Monza, Italy), and 50 mM NaF was added to all buffers. Homogenates were mixed for 20 min at 4 C and centrifuged 20 min at 4 C at maximum speed. Supernatant was collected and stored at −80 C. The protein content was determined by bicinchoninic acid assay (Pierce, Rockford, IL). For the preparation of hippocampal extracts from P19 mice, tissues were homogenized in RIPA buffer (Tris-HCl, 50 m M, NaCl 150 mM. Triton X-100 1%, SDS, 0,1%, sodium deoxycholate 0,5%, PMSF 1 mM, protease and phosphatase inhibitors cocktail, 1% (Sigma)). Proteins were separated by SDS-PAGE, transferred to nitrocellulose membranes, blocked in 5% milk, TBS, 0.1% Tween, and incubated with rabbit anti-CDKL5 1∶250 (Sigma-Aldrich), mouse anti-Cdkl5 1∶250 (see below), and rabbit anti-MECP2 (1∶1000), anti-AKT (1∶1000), anti-p-AKT (1∶1000), anti-BDNF (1∶200; Santa Cruz Biotechnology, Santa Cruz, CA) and anti-tubulin (1∶5000; Sigma-Aldrich) overnight at 4 C, incubated with secondary antibodies (1 h at room temperature), developed using ECL detection (GE Healthcare, Chalfont St. Giles, UK), and images acquired and quantified using a digital camera (ChemiDoc XRS+ System, BioRad, Hercules, CA).

### Semi-quantitative and quantitative PCR

Brains were collected from mice at 2 months, washed in PBS-DEPC and rapidly frozen in liquid nitrogen. One hemisphere was homogenized using an automated dounce and total RNA extracted (RNeasy Mini Kit, Qiagen, Hilden, Germany) and converted into first-strand cDNA (SuperScript II Reverse Transcriptase, Invitrogen, Paisley, UK) using oligo-dT according to the manufacturer's protocol. For semi-quantitative PCR, DNA was amplified using primers against *Cdkl5* exons using 1× PCR Buffer (Promega, Madison, WI), 0.5 units of Dream Taq (Promega) and 200 mM each dNTPs (Fermentas, Vilnius, Lithuania). For SYBER green qPCR (Finnzymes, Vantaa, Finland) exons were amplified using primers against exons 2–3 and 9–10 and control primers against Abl.

### EEG analysis

Male mice (2–4 months) were anesthetized with ketamine/xylazine supplemented with isofluorane as needed, kept on a heating pad to maintain body temperature at 35±1 C, and immobilized in a stereotaxic frame. An incision above the skull was cut and burr holes drilled into the skull. Four stainless steel screws were used as electrodes placed bilaterally above hippocampus (2.0 mm posterior, 1.5 mm lateral to bregma) and frontal cortex (1.8 mm anterior, 1.5 lateral to bregma). Ground and reference screws were anchored on the posterior and middle portions of the skull, respectively. A wireless Neurologger 2A recording device (400 HZ sampling rate) acquired and stored data in real-time for later downloading [19]. After surgery animals were housed individually and allowed at least 1 week to recover. Mice were tested in a novel cage for a 30 min baseline period followed by a 2 hour recording after treatment with kainic acid (10 mg/kg and 25 mg/kg, i.p.). Each animal received both doses separated by at least one day. Data were downsampled to 200 Hz and filtered between 1–25 Hz (Chebyshev I filter, 3rd order). To quantify seizure episodes, a Fourier transform (4 s window, 3.5 s overlap, 2 hours period) was applied to the EEG. Seizure events in the 1–8 Hz frequency range were used to quantify amplitude. The baseline period was used as a cutoff criterion (mean power+8x SD) to define seizure events.

### Behavior testing

#### Clasping

Mice were suspended by their tail for 2 min and hind-limb clasping was assessed from video recordings. Clasping was defined as present if it occurred for more than 5 seconds in an animal.

#### Home-cage activity

Locomotion was measured using activity-monitoring cages similar in size, shape, and material to the home cage (TSE Systems, Bad Homburg, Germany). A mouse was placed in the chamber at least 3 h before recording started. Relative activity was monitored continuously for four days and binned into 12 h epochs.

#### Open field

Mice were placed in the center of a 50×50 cm open arena equipped with video tracking and infrared rearing detection systems (VideoMot2, TSE Systems). Cumulative distance travelled was collected in 5 min intervals for 30 min.

#### Visual drum

Mice were placed on a 12 cm diameter platform within a 28 cm visual-tracking drum (L&H Creations, Baldenheim, France). The opto-kinetic response (head tracking) was assessed from video recordings. Each mouse was tested using vertical stripes (6 mm and 15 mm width) at two speeds (2 rpm and 4 rpm) and data were averages as genotype effects were similar across conditions.

#### VEPs

Animals were anesthetized with urethane (0.7 ml/kg i.p.; 20% in saline; Sigma-Aldrich) and placed in a stereotaxic frame with full viewing of the visual stimulus. Additional doses of urethane were used, if necessary, to keep the anesthesia level stable throughout the experiment. Body temperature was monitored with a rectal probe and maintained at 37.0 C with a heating pad. A hole was drilled bilaterally in the skull, overlying the binocular portion of the primary visual cortex (binocular area Oc1B). After exposure of the brain surface, the dura was removed. A glass micropipette (4 µm tip, 3 M NaCl) was inserted perpendicularly to the stereotaxis plane into the cortex contralateral to the measured eye. In most experiments, microelectrodes were inserted 3.1–3.3 mm lateral to the intersection between sagittal- and lambdoid-sutures and advanced 100 µm within the cortex. Electrical signals were amplified (5,000–20,000 fold), bandpass filtered (0.3–100 Hz), and averaged (at least 50 blocks of 2 events each) in synchrony with the stimulus contrast reversal. Transient VEPs in response to abrupt contrast reversal (1 Hz) were evaluated by measuring the peak-to-baseline amplitude and peak latency of the major component. VEPs in response to a blank stimulus were also frequently recorded to estimate of noise. Visual stimuli were horizontal sinusoidal gratings of different spatial frequency and contrast generated by a VSG2/2 card (Cambridge Research System, Cheshire, UK) and presented on a computer display (25 cm distance, mean luminance = 25 cd/m^2^). VEP amplitude decreases with increasing stimulus spatial frequency; visual acuity was obtained by extrapolation to zero amplitude of the linear regression through the last four to five data points in a curve where VEP amplitude is plotted against log spatial frequency [20].

### Monoclonal antibody production

Portions of the Cdkl5 cDNA encoding amino acids 13–297 and 766–938 were cloned in the N-terminal His6-tag SUMO3 vector for expression in bacteria followed by His-tag purification and SenP2 cleavage. About 10 µg of purified protein was injected into CD-1 mice to raise antibodies. Seven injections were necessary to obtain a high titer antigen-response as tested by ELISA. Spleen was taken from the immunized mouse and the splenocytes were fused to Mouse myeloma cell-line Sp2 using polyethylene glycol. Fused cells were plated into twenty 96 well plates, with approximately one cell/well. The resulting hybridoma clones were tested for reactivity by antigen microarray against the immobilized original antigens [21]. Six and seven hybridomas were positive for the N-terminal and C-terminal antigens, respectively, and 1/6 and 2/7 clones showed a band of the expected size on Western blots using brain extracts from wild-type mice that were absent from knockout mice. A single clone for each antigen was submitted for large scale IgG purification (InVivo BioTech Services, Hennigsdorf, Germany). The antibody raised against the C-terminal of Cdkl5 was used in the present work.

### Statistical Analysis

Data were analyzed using Student's t-test (for male genotype) as well as one-or two-way ANOVA (for female genotype) using Prism software (GraphPad, La Jolla, CA; α = 0.05). *Post hoc* comparisons were analyzed by two-tailed paired and unpaired t-tests. Dendrite length was analyzed using the Kolmogorov-Smirnov (K-S) fitting test, and then paired comparisons. For immunohistochemistry, data were statistically analyzed by Student's t-test and one or two-way ANOVA using Prism software (GraphPad). For the open field, data were analyzed in 5 min increments using a two-way ANOVA with repeated measures

## Results

### Construction and validation of *Cdkl5* conditional knockout mice

**Figure 1:**
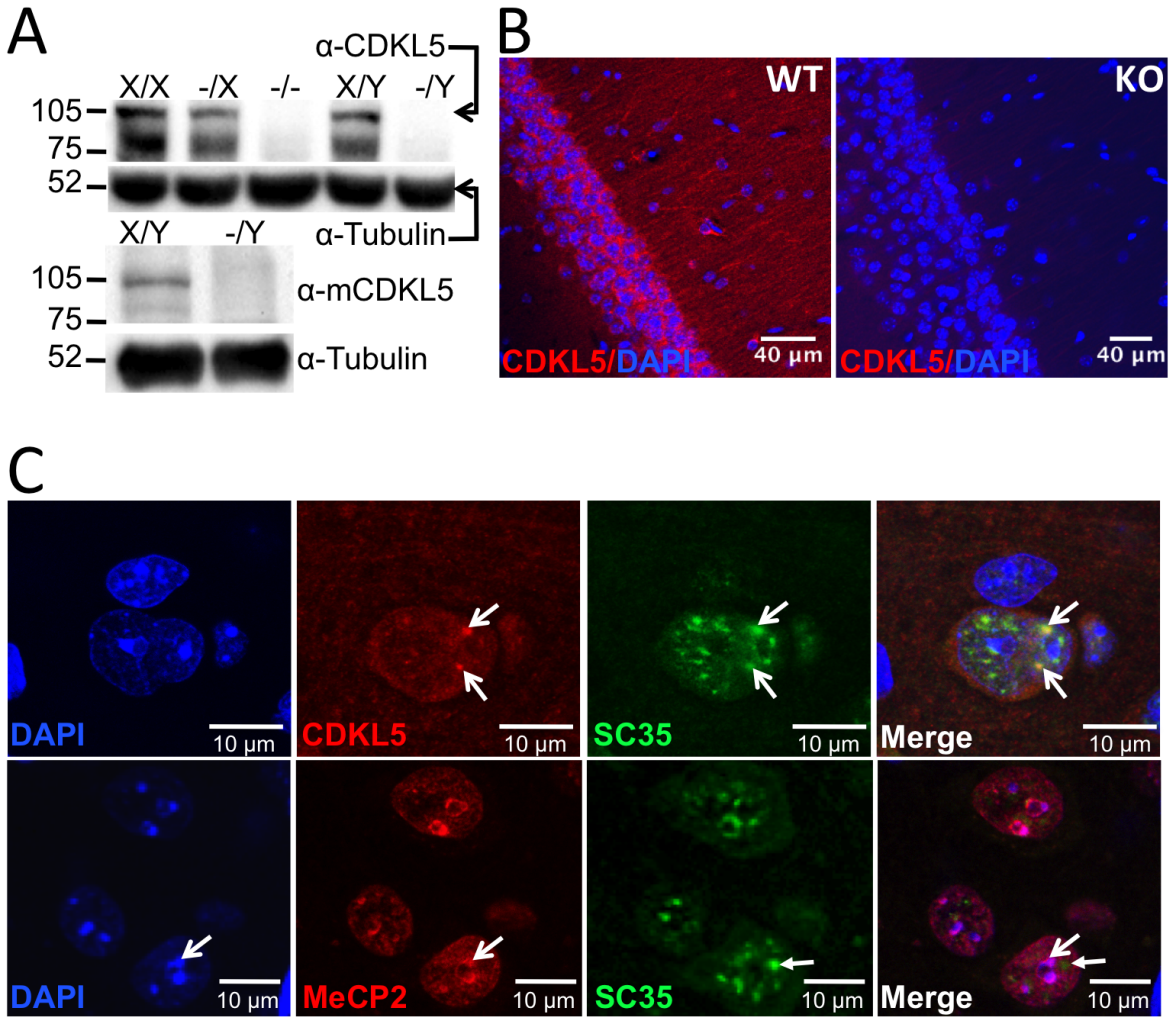
Validation of *Cdkl5* knockout mice. (**A**) Western blot analysis of whole brain protein extracts of wild-type (X/X), heterozygous (-/X), and homozygous (-/-) female and wild-type (X/Y) and hemizygous (-/Y) male *Cdkl5* knockout mice using polyclonal (top panel) and monoclonal (bottom panel) anti-Cdkl5 antibodies. (**B**) Immunofluorescence analysis of CA1 hippocampus brain sections from adult male wild-type (WT) and *Cdkl5* knockout (KO) mice using polyclonal anti-Cdkl5 antibody, showing staining of neuronal cell bodies and nuclear puncta (Scale bar 40 µm). (**C**) Anti-Cdkl5, SC35 and Mecp2 immunofluorescence analysis of S1 cortex brain sections from adult male wild-type (WT) mice. Arrowheads point to regions of co-localization between CDKL5 and SC35 and Mecp2 and SC32 (Scale bar 10 µm).

A constitutive knockout allele of *Cdkl5* was produced by germline deletion of exon 4 of a *Cdkl5* conditional knockout allele produced by standard gene targeting in embryonic stem cells (**Figure S1A**). Western blot analysis of whole brain extracts (**Figure 1A**) and immunofluorescence of brain sections (**Figure 1B**) confirmed the absence of Cdkl5 protein in hemizygous male and homozygous female knockout mice and intermediate levels in heterozygous females. Absence of the full-length protein in whole brain extracts of *Cdkl5* knockout mice was further confirmed by Western blot analysis with a monoclonal antibody (EA7) raised against the C-terminus of mouse Cdkl5 (**Figure 1A**). Immunofluorescence confirmed the localization of Cdkl5 protein to both cytoplasm and nucleus of neurons (**Figure 1BC** **and Figure S1F**) with co-localization in the nucleus with the nuclear speckle marker SC35, as previously reported in cultured cells [10]. Notably, cytoplasmic staining was more prominent in hippocampal than in cortical pyramidal neurons suggesting a cell-type specific regulation of nuclear translocation (**Figure 1BC**). Immunoreactivity was seen in astrocytes, but not microglia as identified by their homogeneous and compact DAPI staining, respectively. On the other hand, nuclear Mecp2 immunoreactivity did not co-localize with SC35, but rather mirrored the pattern of heterochromatin revealed by DAPI staining (**Figure 1C**). These data confirm the distinct nuclear localization of Cdkl5 and Mecp2 in brain and suggest that they have at least partially non-overlapping functions there.

### Behavioral deficits in *Cdkl5* knockout mice

**Figure 2:**
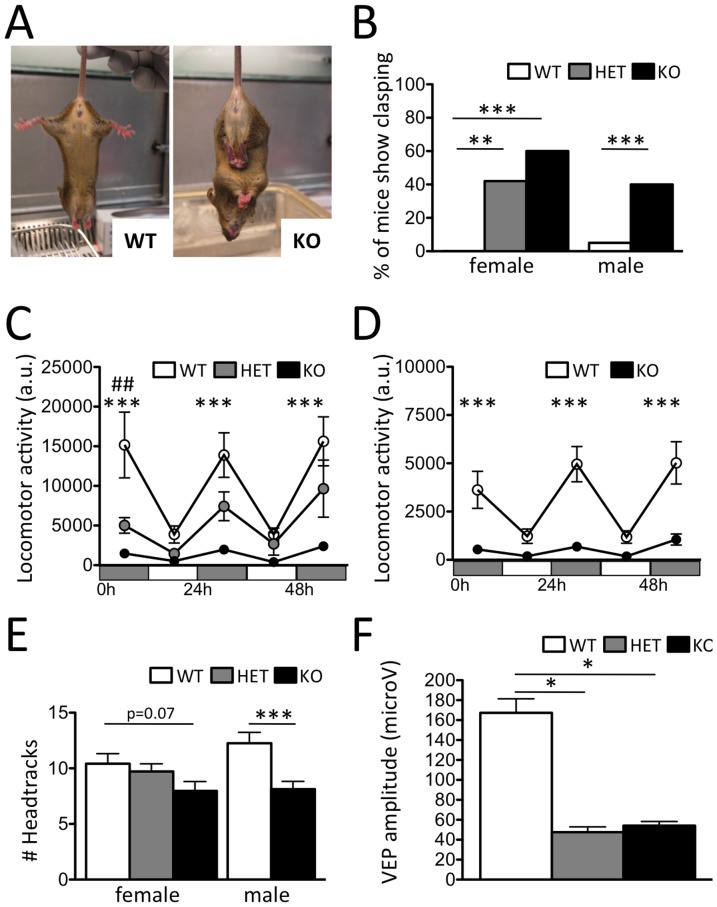
Behavioral impairments in *Cdkl5* knockout mice. (**A**–**B**) Percentage of mice showing hind-limb clasping was significantly increased in adult female and male *Cdkl5* knockout mice (X/X, N = 28; -/X, N = 38; -/-, N = 32; X/Y, N = 55; -/Y, N = 42). Home cage activity was significantly decreased in adult (**C**) female and (**D**) male *Cdkl5* knockout. (**E**) Number of eye tracking saccades was significantly decreased in adult female and male *Cdkl5* knockout mice (X/X, N = 10; -/X, N = 9; -/-, N = 10; X/Y, N = 10; -/Y, N = 11). (**F**) Amplitude of the VEP short latency wave evoked by a low spatial frequency grating (0.05 c/deg) was significantly reduced in adult female *Cdkl5* knockouts (X/X, N = 5; -/X, N = 3; -/-, N = 6; *P<0.05, **P<0.01).

Hemizygous male and homozygous female *Cdkl5* knockout mice showed normal viability, body weight, and absolute as well as relative brain weight (**Figure S2AE**). A general behavioral screen [22] revealed abnormal clasping of hind-limbs in a significant fraction of heterozygous and homozygous female as well as hemizygous male *Cdkl5* knockout mice while no, or very low levels of clasping were seen in wild-type littermates (**Figure 2AB**). Continuous monitoring of home cage activity showed a significant decrease in locomotion in both homozygous female and hemizygous male *Cdkl5* knockouts and intermediate levels in heterozygous *Cdkl5* knockout females when compared to wild-type littermates (**Figure 2CD**). However, hypolocomotion was not seen when mice were placed in a novel open arena (**Figure S2B**) suggesting that the deficit did not reflect a reduced capacity for locomotion. Next, we measured head tracking responses to a continuously moving visual stimulus in the visual drum test [23]. Hemizygous male *Cdkl5* knockout mice showed a significant decrease in the number of head tracks performed in the test compared to wild-type littermates while homozygous female knockouts showed a trend for reduced head tracking (**Figure 2E**). To determine whether head tracking deficits might be a consequence of defects in visual system function we quantified visual evoked potentials (VEPs) [24] to estimate visual acuity. The amplitude, but not latency, of the first positive wave (recording from V1 superficial layers) was significantly reduced in both heterozygous and homozygous knockout females when compared to wild-type littermates (**Figure 2F**) indicating deficient visual processing in the mutant mice.

**Figure 3:**
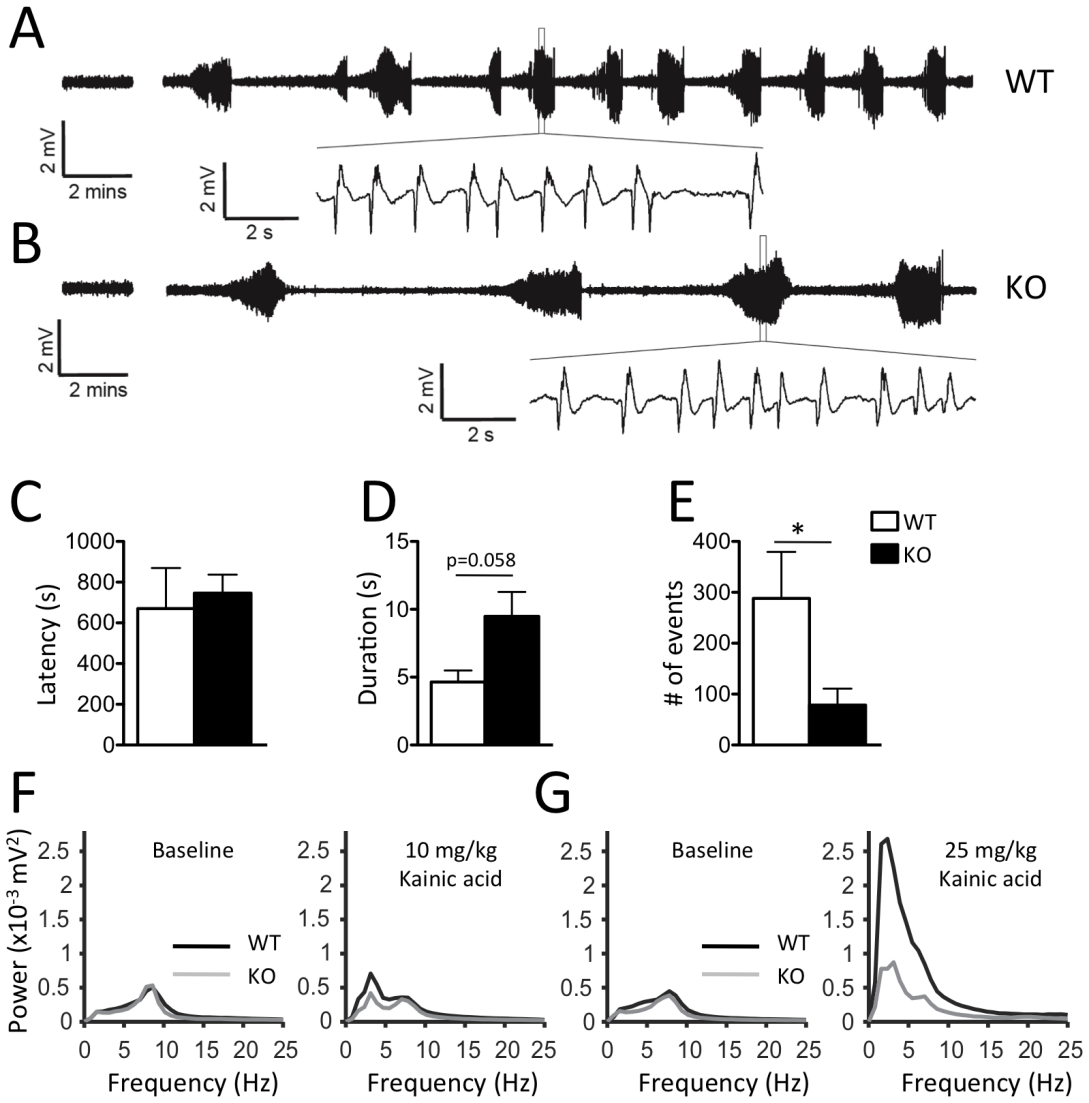
Altered seizure response in *Cdkl5* knockout mice. (**A–B**) Representative electroencephalogram (EEG) traces recorded from surface electrodes placed over the somatosensory cortex in freely moving male wild-type (WT) and *Cdkl5* knockout (KO) mice. (**Left**) Baseline EEG before drug treatment. (**Right**) EEG taken during 2 hour post-injection period following treatment with high dose (25 mg/kg, i.p.) kainic acid. (**expanded trace**) Detail of epileptiform event showing low frequency, high amplitude activity. (**C**) Latency to the first epileptiform event did not differ between wild-type and *Cdkl5* knockout mice, but (**D**) mean duration of events was longer and (**E**) mean frequency was lower in knockouts. Average EEG power spectra of (**left**) baseline and (**right**) post-injection periods for (**F**) low dose (10 mg/kg, i.p.) and (**G**) high dose (25 mg/kg, i.p.) kainic acid treatment revealed a significant, dose-dependent increased in low frequency EEG power in wild-type, but not *Cdkl5* knockout mice (mean ± SEM; WT: N = 4, KO: N = 5).

Although early onset seizures are a prominent feature of CDKL5 disorder, no evidence for spontaneous seizures emerged during videotaped observations of adult *Cdkl5* knockout mice either in the home cage or following transfer to a novel cage. Electroencephalographic (EEG) recordings from implanted surface electrodes in freely behaving animals did not reveal spontaneous epileptiform activity in hemizygous male *Cdkl5* knockout mice (**Figure 3AB**). Pharmacological induction of seizures with kainic acid was monitored by surface EEG. Low dose kainic acid did not induce overt seizures, but caused occasional epileptiform activity patterns in both hemizygous *Cdkl5* male knockouts and wild-type littermates. At the higher dose, kainic acid induced overt seizures, as evidenced by periods of sudden immobility and in some cases tonic-clonic convultions in both hemizygous *Cdkl5* knockout and wild-type littermate mice. Correspondingly, prominent epileptiform activity bursts were observed in the EEG of both genotypes (**Figure 3AB**). *Cdkl5* knockout mice did not differ from wild-type littermates in latency to epileptiform activity bursts suggesting similar susceptibility to the drug (**Figure 3C**). However, the mean duration of high-amplitude bursts was longer and the frequency lower in *Cdkl5* knockout compared to wild-type littermates (**Figure 3DE**). Power spectrum analysis revealed a significant dose-dependent increase in low frequency EEG power in wild-type, but not hemizygous *Cdkl5* male knockouts treated with kainic acid when compared to baseline (**Figure 3FG**). To investigate whether genetic background might alter the penetrance or expressivity of the *Cdkl5* mutation on EEG activity, we backcrossed consitutive *Cdkl5* knockout mice onto the DBA/2J background. Consistent with previous reports [25], seizure susceptibility was signficantly enhanced on this genetic background as evidenced by more severe seizures with the same kainic acid dose (data not shown). However, no evidence of spontaneous seizures emerged in the knockout mice and kainic acid-induced epileptiform activity was similar in wild-type and *Cdkl5* knockout mice on this genetic background (**Figure S3**) suggesting that the relative seizure resistence of the founder background was not masking an epileptic phenotype in the knockout. These data reveal that while *Cdkl5* knockout mice do not exhibit spontaneous seizures or increased seizure susceptibility, they do show abnormal EEG response to pro-convulsant treatment.

### Reduced dendritic arborization in *Cdkl5* knockout mice

**Figure 4:**
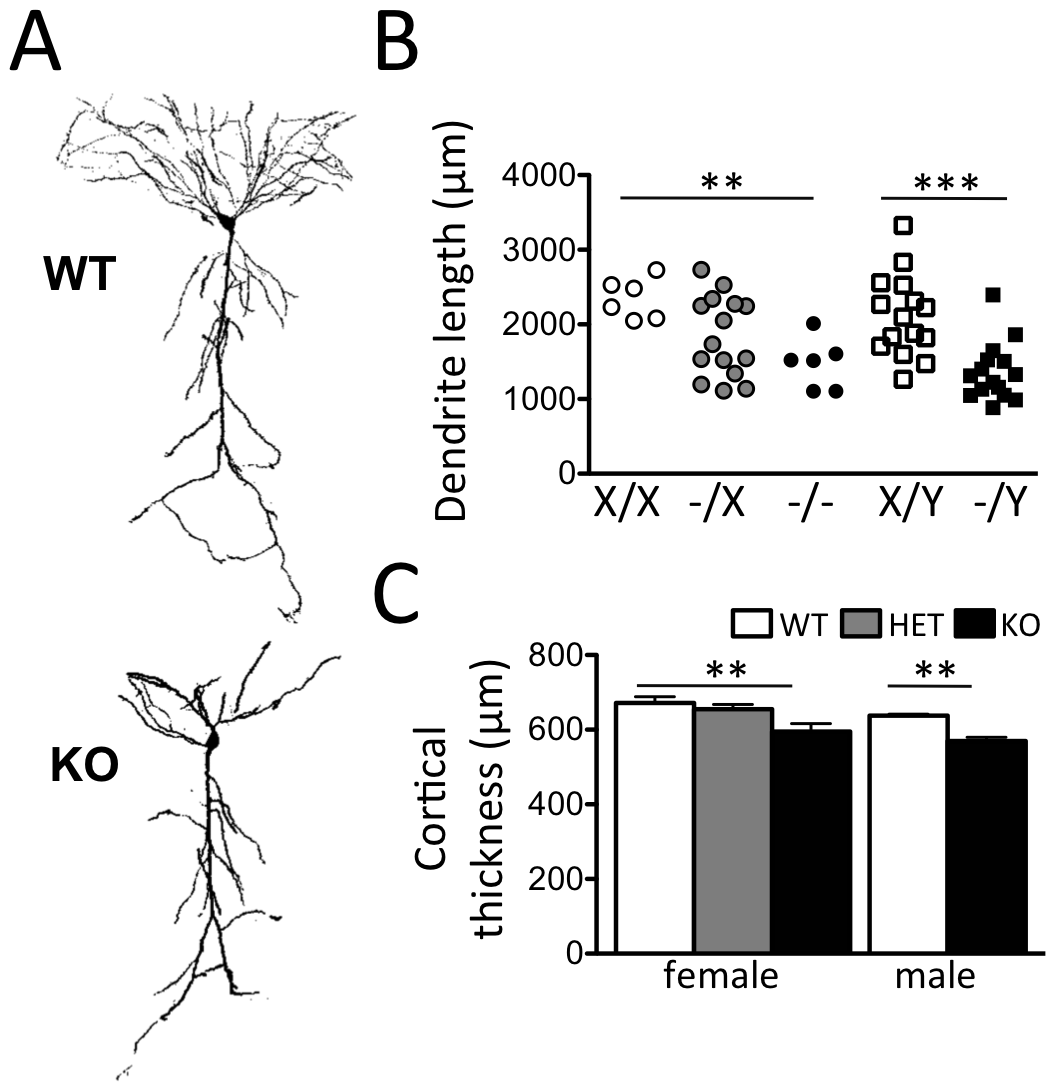
Abnormal dendritic branching in *Cdkl5* knockout mice. (**A**) Representative images of reconstructed neurons from adult male wild-type (WT, top panel) and *Cdkl5* knockout (KO, bottom panel) mice. (**B**) Total dendrite length was significantly reduced in female and male *Cdkl5* knockout mice (X/X, N = 6; -/X, N = 15; -/-, N = 6; X/Y, N = 15; -/Y, N = 15). Heterozygous female knockout mice showed a bimodal distribution (K–S test, P = 1.2×10^−14^). (**C**) Significantly reduced cortical thickness was observed in *Cdkl5* knockout compared with WT controls in female and male mice (X/X, N = 3; -/X, N = 3; -/-, N = 3; X/Y, N = 3; -/Y, N = 3; mean ± SEM, *P<0.05, **P<0.01, ***P<0.001).

Dendritic arborization is significantly reduced in cortical pyramidal neurons from both Rett Syndrome subjects [15], [26], [27] as well as *Mecp2* knockout mice [28], [29]. It is not know if similar deficits exist in the brains of subjects carrying *CDKL5* mutations. However, siRNA-mediated knockdown of Cdkl5 causes a reduction in dendritic arborization both *in vitro* and *in vivo* [11]. To quantify dendritic arborization we crossed *Cdkl5* knockout mice to mice carrying the *Thy1*::*GFP* transgene [30] and reconstructed individual layer 5 cortical (**Figure 4A**) and CA1 (**Figure S4 G,H**) pyramidal neurons. Total length of apical dendritic arbors was significantly reduced in homozygous female and hemizygous male *Cdkl5* knockout cortical and hippocampal pyramidal neurons compared to wild-type littermates, while dendritic arbor length of cortical neurons in heterozygous female *Cdkl5* knockout mice showed an intermediate mean distribution (**Figure 4B** **and S4I**). Notably, dendritic arbor length in heterozygous female mice showed a bimodal distribution (Kolmogorov-Smirnov test, P = 1.2×10^−14^) consistent with a cell autonomous function of the X-linked *Cdkl5* gene in cells in which either one or the other X chromosome has been inactivated. Reduced dendritic arborization was associated with a significant reduction in cortical thickness in both homozygous female and hemizygous male *Cdkl5* knockout mice when compared to wild-type littermates with intermediate levels seen in heterozygous female knockouts (**Figure 4C**). Significant reductions in the thickness of hippocampal layers were found, including CA1 *stratum oriens* and the molecular layer of both the upper and lower blades of the dentate gyrus (**Figure S4B–F**). Sholl analysis of pyramidal neuron dendrites revealed significant decreases in branching at 100–130 µm from the soma of cortical pyramidal neurons and at 80–120 µm and 140–160 µm from the soma of hippocampal pyramidal neurons in homozygous female and hemizygous male *Cdkl5* knockout mice compared to wild-type littermates (**Figure S5AB**; P<0.05, Tukey test).

### Signaling deficits in *Cdkl5* knockout mice

**Figure 5:**
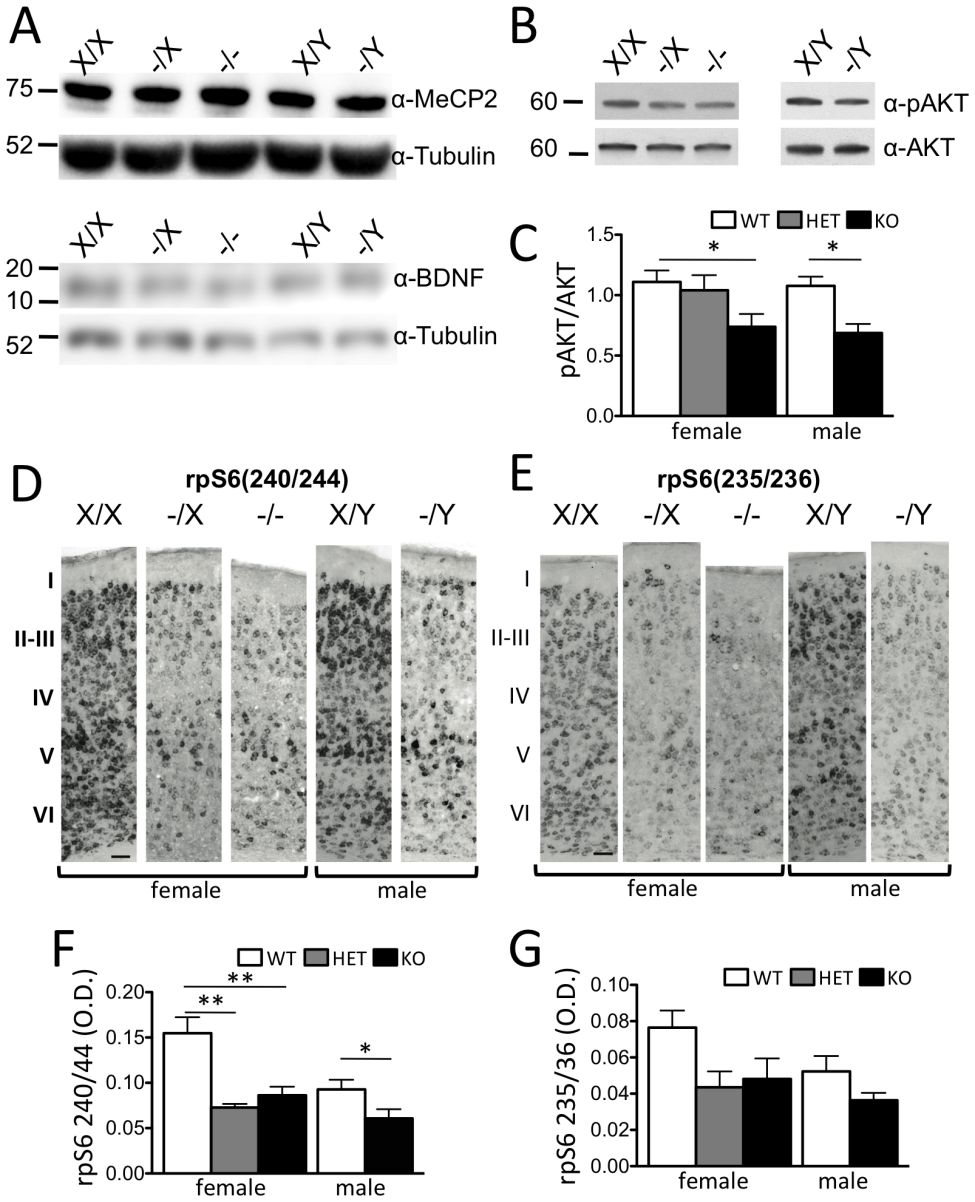
Defective cellular signalling in *Cdkl5* knockout mice. (**A**) Western blot analysis of whole brain protein extracts from adult female and male wild-type and *Cdkl5* mutant mice. Tubulin was included as a loading control. No change was seen in MeCP2 (top panel) and BDNF (lower panel) levels in mutant *Cdkl5* mice compared with wild-type controls (X/X, N = 10; -/X, N = 6; -/-, N = 10; X/Y, N = 5; -/Y, N = 6). (**B**) Western blot analysis of hyppocampal protein extracts from P19 female and male wild-type and *Cdkl5* mutant mice. Decreased pAKT was observed in Cdkl5 mutant mic4 compared with wild-type controls (X/X, N = 4; -/X, N = 6/8; -/-, N = 6; X/Y, N = 3; -/Y, N = 5. (**C**) Western blot quantification revealed a significant decrease in phospho-Akt immunoreactivity protein in mutant *Cdkl5* mice compared with wild-type controls (female: WT, N = 10, HET, N = 6, KO, N = 10; male: WT, N = 5, KO, N = 6; mean ± SEM) (mean ± SEM; *P<0.05, **P<0.01). (**D**) Representative anti-phospho-rpS6 (240/244) immunohistochemistry in the S1 cortex of adult wild-type and *Cdkl5* mutant mice. (E) Representative anti-phospho-rpS6 (235/236) immunohistochemistry in the S1 cortex of adult wild-type and *Cdkl5* mutant mice. A significant reduction of phosphorylation at serine 240/244 (**F**) and a trend for reduced phosphorylation at serine 235/236 (**G**) of rpS6 was observed in layer II–III and V of male and female mutant mice compared to wild-type controls (X/X, N = 4; -/X, N = 6; -/-, N = 3; X/Y, N = 4; -/Y, N = 4; *P<0.05, *P<0.01).

Next, we examined whether expression of Mecp2 protein and signaling factors known to be altered in *Mecp2* knockout mice might be similarly affected in *Cdkl5* knockouts. Western blots on whole brain extracts revealed no change in Mecp2 protein levels in *Cdkl5* knockout mice (**Figure 5A** and **Figure S6A**). Levels of BDNF immunoreactivity, reported to be reduced in *Mecp2* knockout brain [31]–[33], were unaltered in *Cdkl5* knockout brain (**Figure 5A** and **Figure S6B**). However, decreased levels of phosphorylated Akt were observed in extracts of hippocampus from *Cdkl5* knockouts when compared to wild-type littermates (**Figure 5BC**). Moreover, as reported in *Mecp2* mutant mice [18], levels of ribosomal protein S6 (rpS6) phosphorylated at position 240/244 were significantly reduced in somatosensory cortex of homozygous and heterozygous female and hemizygous male *Cdkl5* knockout mice when compared to wild-type littermates (**Figure 5DF**). Levels of rpS6 phosphorylated at position 235/236 showed a trend for reduction in mutants (**Figure 5EG**). An analysis of individual cortical layers revealed reduced levels of phospho-rpS6 (240/244) and phospho-rpS6 (235/236) across layers 2–6 in both female and male mutant mice (**Figure S7C–F**).

### Mapping of *Cdkl*5 behavioral phenotypes

**Figure 6:**
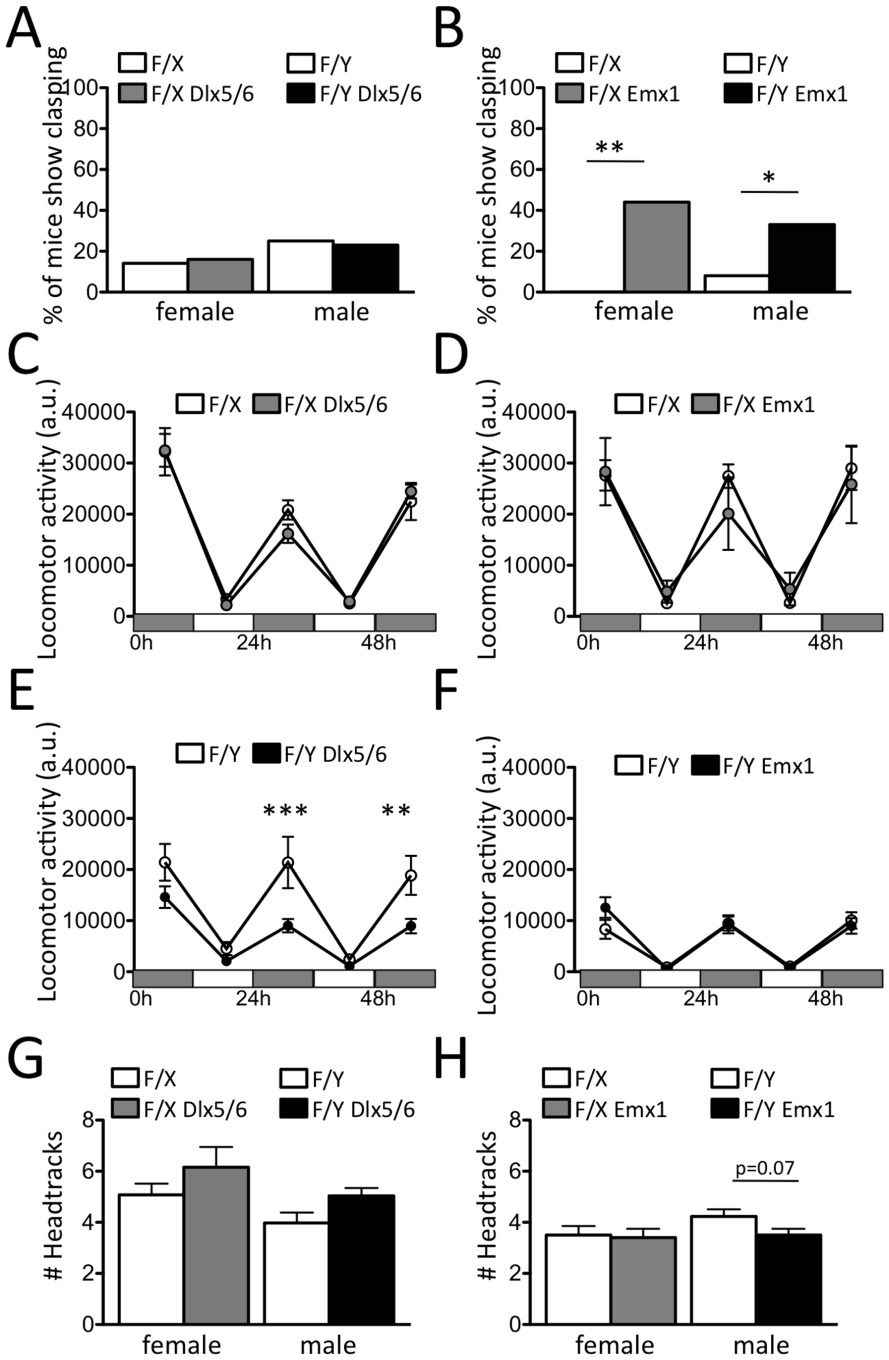
Behavioral impairments in *Cdkl5* conditional knockout mice. (**A**–**B**) Percentage of mice showing hind-limb clasping was significantly increased in heterozygous female and hemizygous male (**B**) *Emx1* (F/X, N = 13; F/X-*Emx1*::Cre, N = 9; F/Y, N = 12; F/Y-*Emx1*::Cre, N = 9), but not (**A**) *Dlx5/6*-conditional *Cdkl5* knockout mice (F/X, N = 7; F/X-*Dlx5/6*::Cre, N = 6; F/Y, N = 4; F/Y-*Dlx5/6*::Cre, N = 13). (**C–F**) Home cage activity was significantly decreased in (**E**) male hemizygous (F/Y, N = 6; F/Y-*Dlx5/6*::Cre, N = 13), but not (**C**) female heterozygous (F/X, N = 5; F/X-*Dlx5/6*::Cre, N = 4) *Dlx5/6*-conditional *Cdkl5* knockout mice when compared to control littermates. Normal locomotion activity was observed in (**D**) female and (**F**) male *Emx1*-conditional *Cdkl5* knockout mice when compared to control littermates (F/X, N = 12; F/X-*Emx1*::Cre, N = 6; F/Y, N = 15; F/Y-*Emx1*::Cre, N = 9). (**G–H**) Number of head tracking saccades was decreased in male, but not female *Emx1*-conditional *Cdkl5* knockout mice (F/Y, N = 7; F/Y-*Emx1*::Cre, N = 6; F/X, N = 5; F/X-*Emx1*::Cre, N = 6). No evidence for deficient head tracking was observed in *Dlx5/6*-conditional *Cdkl5* knockout mice (F/Y, N = 5; F/Y-*Dlx5/6*::Cre, N = 11; F/X, N = 5; F/X-*Dlx5/6*::Cre, N = 5; ***P<0.001, **P<0.01, *P<0.05).

To help identify the cell-types in which Cdkl5 deletion drives pathological phenotypes, we examine mice carrying a Cre-conditional knockout (cKO) allele of *Cdkl5* (**Figure S1A**). For deletion in forebrain GABAergic neurons (e.g. cortical interneurons, striatal medium spiny neurons) we crossed the *Cdkl5* conditional knockout allele with the *Dlx5/6*::Cre transgene [34]. For deletion in cortical glutamatergic neurons (e.g. cortical and hippocampal pyramidal neurons) we crossed the *Cdkl5* conditional knockout allele with the *Emx1*::Cre transgene [35]. Both *Dlx5/6* and *Emx1* conditional knockout mice appeared outwardly normal at birth and showed normal body weight and viability when compared to littermate controls (**Figure S8**). A general behavioral screen [22] revealed abnormal clasping of hind-limbs in a significant fraction of heterozygous female as well as hemizygous male *Emx1*-conditional, but not *Dlx5/6*-conditional *Cdkl5* knockout mice when compared to control littermates (**Figure 6AB**). Continuous monitoring of home cage activity revealed a significant decrease in locomotion in hemizygous male *Dlx5/6*-conditional, but not *Emx1*-conditional *Cdkl5* knockouts (**Figure 6EF**) when compared to control littermates. Only a trend for reduced locomotion was seen in heterozygous *Dlx5/6*-conditional *Cdkl5* knockout females (**Figure 6CD**) consistent with a dose-dependent effect of *Cdkl5* on this phenotype (**Figure 2C**). Next, we measured head tracking responses to a continuously moving visual stimulus in the visual drum test [23]. Hemizygous male, but not heterozygous female, *Emx1*-conditional *Cdkl5* knockout mice showed a trend (P = 0.07) for decreased head tracking compared to control littermates (**Figure 6H**), while *Dlx5/6*-conditional knockouts did not show deficits in head tracking (**Figure 6G**). Taken toghether these data argue that the behavioral phenotypes seen in *Cdkl5* knockouts can be mapped to diverse forebrain neuronal populations, with defects in limb clasping and head tracking associated with glutamatergic neurons and hypolocomotion associated with GABAergic neurons.

## Conclusions

Constitutive *Cdkl5* knockout mice recapitulate several core features of CDKL5 disorder and serve as a useful animal model of the disorder. In particular, *Cdkl5* knockout mice showed hind-limb clasping, hypoactivity, defective head tracking, and abnormal EEG responses to convulsants (**Figure 2** **–** **3**), features that may model the stereotypic hand movements, hypotonia, eye-tracking abnormalities, and seizures, respectively, reported in the human condition. Although early-onset seizures are a key feature of CDKL5 disorder, we failed to detect any spontaneous seizure or epileptiform activity in mutant mice on two different genetic backgrounds (**Figure 3** and **S3**). We interpret these findings as an indication that Cdkl5 impacts on the development of epilepsy in humans in a manner significantly different from mice. However, our findings of an altered profile of EEG burst activity following kainic acid treatment of *Cdkl5* knockout and wild-type mice (**Figure 3** **and S3**), may nevertheless be useful to identify epileptic mechanisms impacted by Cdkl5 in the human condition.

The phenotype of *Cdkl5* knockout mice partially overlaps with that of *Mecp2* knockouts. Both mutants showed hind-limb clasping, hypoactivity, and reduced dendritic branching of cortical neurons. In addition, cortical neurons in both knockouts showed reduced phosphorylation of rpS6, a ribosomal regulatory subunit and modulator of protein translation [36]–[38], and Akt, a critical component of neurotrophin signaling [39] and up-stream modulator of rpS6 via mTOR [38]. These findings suggest that downregulation of the rpS6 pathway may be a common signaling deficit in CDKL5 disorder and Rett Syndrome and point to defective translational regulation as a potential core mechanisms for common pathological features of the disorder. Importantly, no evidence emerged for altered BDNF expression or function in *Cdkl5* knockout mice (**Figure 5A** **and S6B**) suggesting that this pathways may not play a major role in common features of CDKL5 disorder and Rett Syndrome.

The non-overlapping intracellular localization of Cdkl5 and Mecp2 proteins further argues that they act via different signaling mechanisms to affect common targets. While Mecp2 is nuclear and associated with heterochromatin [10] (**Figure 1C**), Cdkl5 is both cytoplasmic and nuclear, in the latter case being associated with speckles, granular structures containing splicing proteins [10]. Although Cdkl5 function within nuclear speckles is presently unknown, downregulation of Cdkl5 has been shown to increase speckle size in cultured cells [10]. On the other hand, current evidence suggests that Mecp2 acts as a chromatin-associated transcriptional repressor to alter cellular gene expression and modify neuronal function via the secretion of extracellular factors [40].

Conditional knockout of *Cdkl5* in glutamatergic cortical neurons (with *Emx1*::Cre) and GABAergic forebrain neurons (with *Dlx5/6*::Cre) revealed a double dissociation of behavioral phenotypes (**Figure 6**). These findings have several implications. First, they suggest that behavioral deficits in CDKL5 disorder derive from the localized absence of the kinase in forebrain neurons. Second, they suggest that the limb control and eye tracking phenotypes depend on cortical motor and visual circuit deficits that are separable from those underlying hypotonia. Whether the later phenotype depends on deficits in cortical interneuron function or deficits in sub-cortical circuits cannot be determined at this point, although the double dissociation observed in *Emx1*- and *Dlx5/6*-conditional mice suggests that non-cortical regions control the hypolocomotion phenotype. Our findings indicate that therapies aimed at re-expression of CDKL5 in cortical pyramidal neurons may have success in reversing the most debilitating behavioral phenotypes of the disorder.

Several of our findings are similar to those recently described for a constitutive knockout allele of *Cdkl5* in male mice [41]. The hyperlocomotion described in their hemizygous knockout males in a novel environment is similar to the locomotor behavior we see in the open field test (**Figure S2B**), but constrasts with the marked hypolocomotion we see in a familiar environment (**Figure 2D**), a behavior not investigated in their study. The absence of spontaneous seizures and EEG abnormalities is consistent with our results, and the deficits reported in auditory-evoked potentials are complementary to our findings of abnormal visual-evoked potentials (**Figure 2F**). Finally, their data showing a decrease phorphorylation of both Akt and mTOR are consistent with our findings indicating that Akt and rpS6 are hypofunctional (**Figure 5BC and 5F**). Our results extend these findings to females, the primary carriers of pathological CDKL5 alleles, expand the pathological phenotypes involved, and map them to specific cell-types in the brain. In summary, *Cdkl5* knockout mice promise to serve as a useful tool to identify candidate pathological mechanisms and test therapeutic interventions for CDKL5 disorder. Similarities between *Mecp2* and *Cdkl5* knockout mice suggest that core symptoms of the human disorders arise from overlapping cellular defects, while differences may provide clues to the unique pathological deficits associated with CDKL5 disorder.

## Supporting Information

Figure S1 **Generation and validation of** ***Cdkl5*** **knockout mice.** (**A**) Genomic organization of the *Cdkl5* locus showing critical exon 4 (ENSMUSE00000346596), the targeting construct, successfully targeted *Cdkl5* locus (genotyping primers indicated by arrows), FRT-deleted conditional *Cdkl5* knockout allele, and Cre-deleted constitutive *Cdkl5* knockout allele used in the present study. (**B**) Confirmation of homologous recombinants by long-range PCR at 5′end (top panel) and 3′end (bottom panel). PCR products of positive clones (lane 1 and 2) showed specific signals when hybridized with 5′ and 3′ radioactive-labelled DNA probes. No signal was detected in PCR-negative samples, consistent with them being non-homologous or unmodified (lane 3 and 4). (**C**) mRNA expression of *Cdkl5* exons as estimated by Affymetrix microarray hybridization in wild-type and *Cdkl5* knockout brain confirmed absence of the deleted exon 4 in knockout mice, but normal expression of remaining exons consistent with an escape from nonsense-mediated decay. (**D**) Semi-quantitative PCR on total brain RNA from female wild-type and *Cdkl5* mutant mice with primers spanning exons confirmed an absence of exon 4, but normal levels of exons 2, 3, and 5 in mutant mice. (**E**) Quantitative real-time PCR on total brain RNA from female wild-type and *Cdkl5* knockout mice revealed normal levels of expression of upstream (exon 2–3) and downstream (exon 9–10) *Cdkl5* exons. (**F**) Anti-Cdkl5 and SC35 immunofluorescence analysis of S1 cortex brain sections from adult male knockout (KO) mice. (Scale bar 10 µm).
(TIF)

Figure S2 **Normal body and brain weight and novelty-induced locomotion in** ***Cdkl5*** **knockout mice.** (**A**) No difference was observed in body weight of female and male wild-type and *Cdkl5* mutant mice (6 weeks old). (**B**) No difference was detected in total distance travelled in a novel open arena by adult female and male wild-type and *Cdkl5* mutant mice (female: WT, N = 10, HET, N = 9, KO, N = 10; male: WT, N = 10, KO, N = 11). (**C**) Representative images of dissected brains from female wild-type, heterozygous, and homozygous *Cdkl5* knockout mice. (**D**) No difference in relative brain to body weight was detected between genotypes (WT, N = 6, HET, N = 8, KO, N = 7; mean ± SEM). (**E**) Normal viability was observed of female and male wild-type and *Cdkl5* mutant mice (at 6 months of life) (female: WT, N = 48/50, HET, N = 24/27, KO, N = 17/20; male: WT, N = 34/39, KO, N = 51/54, mean ± SEM).
(TIF)

Figure S3 **Seizure response in** ***Cdkl5*** **knockout mice backcrossed to DBA genetic background.** Representative electroencephalogram (EEG) traces recorded from surface electrodes placed over the somatosensory cortex in freely moving male (**A**) wild-type (WT) and (**B**) *Cdkl5* knockout (KO) mice following 3 generations backcrossing to the DBA2/J strain. Power spectra of EEG recordings showed decreased power in *Cdkl5* knockouts when compared to wild-type littermates at low frequencies both under (**C**) baseline conditions and (**D**) in mice injected with kainic acid (25 mg/kg, i.p.; mean ± SEM; WT: N = 3, KO: N = 3).
(TIF)

Figure S4 **Reduced thickness of cortical and hippocampal layers in** ***Cdkl5*** **knockout mice.** (**A**) Representative image showing region of S1 cortex used for quantification of cortical thickness in wild-type and *Cdkl5* mutant mice. (**B**) Representative image showing features used for quantification of hippocampal layer thickness in wild-type and *Cdkl5* mutant mice. (**C–F**) A significant decrease in thickness was observed in hippocampal CA1 *stratum oriens* (but not *stratum laconosum* or *radiatum*) and the lower and upper blades of dentate gyrus molecular layer in female and male *Cdkl5* mutant mice compared to wild-type littermates (female: WT, N = 3, HET, N = 3, KO, N = 3; male: WT, N = 3, KO, N = 3; N = 9–13 sections for each genotype) (mean ± SEM; *P<0.05, **P<0.01). (**G,H**) Representative images of reconstructed neurons from adult male wild-type (WT, G) and *Cdkl5* knockout (KO, **H**) mice. (**I**) Total dendrite length was significantly reduced in male *Cdkl5* knockout mice (X/Y, N = 15; -/Y, N = 15, mean ± SEM, *P<0.05, **P<0.01, ***P<0.001).
(TIF)

Figure S5 **Reduced dendrite complexity in** ***Cdkl5*** **knockout mice.** Classical Sholl analysis with radii increasing in 20 µm increments. Sholl analysis measures apical dendrite length within each sphere plotted against radius from soma. Significant decreases in the numbers of intersections between the dendrites and the Sholl circles from the neuronal somata occurred only between 100 µm and 130 µm in cortical neurons (**A**) and between 80 µm and 120 µm and 140 µm and 160 µm in hyppocampal dendrite (**B**) in mutant *Cdkl5* male mice (Figure S5; P<0.05, Tukey test).
(TIF)

Figure S6 **No change observed in Mecp2 and BDNF levels in** ***Cdkl5*** **knockout mice.** Quantification of western blot data failed to detect a change in immunostaining against (**A**) Mecp2, and (**B**) BDNF protein in mutant *Cdkl5* mice compared with wild-type controls (female: WT, N = 10, HET, N = 6, KO, N = 10; male: WT, N = 5, KO, N = 6; mean ± SEM) (mean ± SEM; *P<0.05, **P<0.01).
(TIF)

Figure S7 **Reduced p-rpS6 protein in** ***Cdkl5*** **knockout mice.** Representative micrographs showing the immunohistochemistry for total rpS6 (**A**) in the S1 cortex of both female and male wild-type and *Cdkl5* mutant mice. Quantitation of immunoreactivity signals revealed no change in total rpS6 protein (**B**) in both male and female mutants, a significant decrease of phospho-rpS6 (240/244) in (**C**) female and (**D**) male mutants, and a decrease of phospho-rpS6(235/236) that was only significant in layer V of (**E**) female mutants, while it shows only a trend in (**F**) male KOs (female: WT, N = 4, HET, N = 6, KO, N = 3; male: WT, N = 4, KO, N = 4; mean ± SEM; *P<0.05, **P<0.01, ***P<0.001).
(TIF)

Figure S8 **Normal body and brain weight** (**A**) No difference was observed in body weight of heterozygous female and hemizygous male (**A**) *Dlx5/6*- (F/X, N = 8; F/X-*Dlx5/6*::Cre, N = 7; F/Y, N = 5; F/Y-*Dlx5/6*::Cre, N = 11) and (**B**) *Emx1* (F/X, N = 12; F/X-*Emx1*::Cre, N = 8; F/Y, N = 12; F/Y-*Emx1*::Cre, N = 10) conditional *Cdkl5* knockout mice at 8 weeks of life. Normal viability was observed at 6 months of life of heterozygous female and hemizygous male (**C**) *Dlx5/6*- (F/X, N = 9/10; F/X-*Dlx5/6*::Cre, N = 6/6; F/Y, N = 5/5; F/Y-*Dlx5/6*::Cre, N = 9/10) and (**D**) *Emx1* (F/X, N = 9/11; F/X-*Emx1*::Cre, N = 5/6; F/Y, N = 11/12; F/Y-*Emx1*::Cre, N = 9/9) conditional *Cdkl5* knockout.
(TIF)

## References

[1] Grosso S, Brogna A, Bazzotti S, Renieri A, Morgese G, et al (2007) Seizures and electroencephalographic findings in CDKL5 mutations: case report and review. Brain Dev 29 (4) 239–242. 17049193 10.1016/j.braindev.2006.09.001

[2] Chahrour M, Zoghbi HY (2007) The story of Rett syndrome: from clinic to neurobiology. Neuron 56: 422–437. 17988628 10.1016/j.neuron.2007.10.001

[3] Fehr S, Wilson M, Downs J, Williams S, Murgia A, et al (2013) The CDKL5 disorder is an independent clinical entity associated with early-onset encephalopathy. Eur J Hum Genet 21 (3) 266–273. 22872100 10.1038/ejhg.2012.156 PMC3573195

[4] Bahi-Buisson N, Nectoux J, Rosas-Vargas H, Milh M, Boddaert N, et al (2008) Key clinical features to identify girls with CDKL5 mutations. Brain 131: 2647–2661. 18790821 10.1093/brain/awn197

[5] Evans JC, Archer HL, Colley JP, Ravn K, Nielsen JB, et al (2005) Early onset seizures and Rett-like features associated with mutations in CDKL5. Eur J Hum Genet 13: 1113–1120. 16015284 10.1038/sj.ejhg.5201451

[6] Russo S, Marchi M, Cogliati F, Bonati MT, Pintaudi M, et al (2009) Novel mutations in the CDKL5 gene, predicted effects and associated phenotypes. Neurogenetics 10 (3) 241–250. 19241098 10.1007/s10048-009-0177-1

[7] Lin C, Franco B, Rosner MR (2005) CDKL5/Stk9 kinase inactivation is associated with neuronal developmental disorders. Hum Mol Genet 24: 3775–3786. 10.1093/hmg/ddi391 16330482

[8] Mari F, Azimonti S, Bertani I, Bolognese F, Colombo E, et al (2005) CDKL5 belongs to the same molecular pathway of MeCP2 and it is responsible for the early-onset seizure variant of Rett syndrome. Hum Mol Genet 14: 1935–1946. 15917271 10.1093/hmg/ddi198

[9] Rusconi L, Salvatoni L, Giudici L, Bertani I, Kilstrup-Nielsen C, et al (2008) CDKL5 expression is modulated during neuronal development and its subcellular distribution is tightly regulated by the C terminal tail. J Biol Chem 283 (44) 30101–30111. 18701457 10.1074/jbc.M804613200 PMC2662074

[10] Ricciardi S, Kilstrup-Nielsen C, Bienvenu T, Jacquette A, Landsberger N, et al (2009) CDKL5 influences RNA splicing activity by its association to the nuclear speckle molecular machinery. Hum Mol Genet 18 (23) 4590–4602. 19740913 10.1093/hmg/ddp426

[11] Chen Q, Zhu YC, Yu J, Miao S, Zheng J, et al (2010) CDKL5, a Protein Associated with Rett Syndrome, Regulates Neuronal Morphogenesis via Rac1 Signaling. J Neurosci 30 (38) 12777–12786. 20861382 10.1523/JNEUROSCI.1102-10.2010 PMC6633570

[12] Chen RZ, Akbarian S, Tudor M, Jaenisch R (2001) Deficiency of methyl-CpG binding protein-2 in CNS neurons results in a Rett-like phenotype in mice. Nat Genet 27 (3) 327–331. 11242118 10.1038/85906

[13] Guy J, Hendrich B, Holmes M, Martin JE, Bird A (2001) A mouse Mecp2-null mutation causes neurological symptoms that mimic Rett syndrome. Nature Genetic 27 (3) 322–326. 10.1038/85899 11242117

[14] Pelka GJ, Watson CM, Radziewic T, Hayward M, Lahooti H, et al (2006) Mecp2 deficiency is associated with learning and cognitive deficits and altered gene activity in the hippocampal region of mice. Brain 129: 887–898. 16467389 10.1093/brain/awl022

[15] Armstrong DD, Dunn JK, Schultz RJ, Herbert DA, Glaze DG, et al (1999) Organ growth in Rett syndrome: a postmortem examination analysis. Pediatr Neurol 20: 125–129. 10082341 10.1016/s0887-8994(98)00124-6

[16] Farley FW, Soriano P, Steffen LS, Dymecki SM (2000) Widespread Recombinase Expression Using FLPeR (Flipper) Mice. Genesis 28: 106–110. 11105051

[17] Tang SH, Silva FJ, Tsark WM, Mann JR (2002) A Cre/loxP-deleter transgenic line in mouse strain 129S1/SvImJ. Genesis 32 (3) 199–202. 11892008 10.1002/gene.10030

[18] Ricciardi S, Boggio EM, Grosso S, Lonetti G, Forlani G, et al (2011) Reduced AKT/mTOR signaling and protein synthesis dysregulation in a Rett syndrome animal model. Hum Mol Genet 20 (6) 1182–1196. 21212100 10.1093/hmg/ddq563

[19] Brankack J, Kukushka VI, Vyssotski AL, Draguhn A (2010) EEG gamma frequency and sleep-wake scoring in mice: Comparing two types of supervised classifiers. Brain Res 1322: 59–71. 20123089 10.1016/j.brainres.2010.01.069

[20] Pizzorusso T, Berardi N, Rossi FM, Viegi A, Venstrom C, et al (1999) TrkA activation in the rat visual cortex prevents the effect of monocular deprivation. Eur J Neurosci 11: 204–212. 9987024 10.1046/j.1460-9568.1999.00417.x PMC2710099

[21] De Masi F, Chiarella P, Wilhelm H, Massimi M, Bullard B, et al (2005) High throughput production of mouse monoclonal antibodies using antigen microarrays. Proteomics 5 (16) 4070–81. 16254927 10.1002/pmic.200401279

[22] Rogers DC, Fisher EMC, Brown SDM, Peters J, Hunter AJ, et al (1999) Behavioral and functional analysis of mouse phenotype: SHIRPA, a proposed protocol for comprehensive phenotype assessment. Mammalian Genome 8: 711–713. 10.1007/s003359900551 9321461

[23] Thaung C, Arnold K, Jackson IJ, Coffey PJ (2002) Presence of visual head tracking differentiates normal sighted from retinal degenerate mice. Neurosci Lett 325 (1) 21–24. 12023058 10.1016/s0304-3940(02)00223-9

[24] Pizzorusso T, Fagiolini M, Porciatti V, Maffei L (1997) Temporal aspects of contrast visual evoked potentials in the pigmented rat: effect of dark rearing. Vision Res 37 (4) 389–395. 9156170 10.1016/s0042-6989(96)00172-1

[25] Schauwecker PE (2011) The relevance of individual genetic background and its role in animal models of epilepsy. Epilepsy Research 97: 1–11. 22001434 10.1016/j.eplepsyres.2011.09.005 PMC3215836

[26] Belichenko NP, Belichenko PV, Li HH, Mobley WC, Francke U (2008) Comparative study of brain morphology in Mecp2 mutant mouse models of Rett syndrome. J Comp Neurol 508 (1) 184–195. 18306326 10.1002/cne.21673

[27] Belichenko NP, Belichenko PV, Mobley WC (2009) Evidence for both neuronal cell autonomous and nonautonomous effects of methyl-CpG-binding protein 2 in the cerebral cortex of female mice with Mecp2 mutation. Neurobiol Dis 34 (1) 71–77. 19167498 10.1016/j.nbd.2008.12.016

[28] Kishi N, Macklis JD (2005) Dissecting MECP2 function in the central nervous system. J Child Neurol 20: 753–759. 16225831 10.1177/08830738050200091001

[29] Stuss DP, Boyd JD, Levin DB, Delaney KR (2012) MeCP2 mutation results in compartment-specific reductions in dendritic branching and spine density in layer 5 motor cortical neurons of YFP-H mice. PLoS One 7 (3) 10.1371/journal.pone.0031896 PMC3296699 22412847

[30] Feng G, Mellor RH, Bernstein M, Keller-Peck C, Nguyen QT, et al (2000) Imaging neuronal subsets in transgenic mice expressing multiple spectral variants of GFP. Neuron 28: 41–51. 11086982 10.1016/s0896-6273(00)00084-2

[31] Zhou Z, Hong EJ, Cohen S, Zhao WN, Ho HY, et al (2006) Brain-specific phosphorylation of MeCP2 regulates activity-dependent Bdnf transcription, dendritic growth, and spine maturation. Neuron 52: 255–269. 17046689 10.1016/j.neuron.2006.09.037 PMC3962021

[32] Chang Q, Khare G, Dani V, Nelson S, Jaenisch R (2010) The disease progression of Mecp2 mutant mice is affected by the level of BDNF expression. Neuron 49: 341–348. 10.1016/j.neuron.2005.12.027 16446138

[33] Lonetti G, Angelucci A, Morando L, Boggio EM, Giustetto M, et al (2010) Early environmental enrichment moderates the behavioral and synaptic phenotype of MeCP2 null mice. Biol Psychiatry 67 (7) 657–665. 20172507 10.1016/j.biopsych.2009.12.022

[34] Zarbalis K, May SR, Shen Y, Ekker M, Rubenstein JL, et al (2004) A focused and efficient genetic screening strategy in the mouse: identification of mutations that disrupt cortical development. PLoS Biol 2 (8) E219. 15314648 10.1371/journal.pbio.0020219 PMC509294

[35] Iwasato T, Nomura R, Ando R, Ikeda T, Tanaka M, et al (2004) Dorsal telencephalon-specific expression of Cre recombinase in PAC transgenic mice. Genesis 38 (3) 130–138. 15048810 10.1002/gene.20009

[36] Pende M, Um SH, Mieulet V, Sticker M, Goss VL, et al (2004) S6K1(−/−)/S6K2(−/−) mice exhibit perinatal lethality and rapamycin-sensitive 5′-terminal oligopyrimidine mRNA translation and reveal a mitogen-activated protein kinase-dependent S6 kinase pathway. Mol Cell Biol 243: 112–124. 10.1128/MCB.24.8.3112-3124.2004 PMC381608 15060135

[37] Ruvinsky I, Meyuhas O (2006) Ribosomal protein S6 phosphorylation: from protein synthesis to cell size. Trends Biochem Sci 31: 342–348. 16679021 10.1016/j.tibs.2006.04.003

[38] Roux PP, Shahbazian D, Vu H, Holz MK, Cohen MS, et al (2007) RAS/ERK signaling promotes site-specific ribosomal protein S6 phosphorylation via RSK and stimulates cap-dependent translation. J Biol Chem 282: 14056–1464. 17360704 10.1074/jbc.M700906200 PMC3618456

[39] Kaplan DR, Miller FD (2000) Neurotrophin signal transduction in the nervous system. Curr Opin Neurobiol 10 (3) 381–391. 10851172 10.1016/s0959-4388(00)00092-1

[40] Guy J, Cheval H, Selfridge J, Bird A (2011) The role of MeCP2 in the brain. Annu Rev Cell Dev Biol 27: 631–652. 21721946 10.1146/annurev-cellbio-092910-154121

[41] Wang IT, Allen M, Goffin D, Zhu X, Fairless AH, et al (2012) Loss of CDKL5 disrupts kinome profile and event-related potentials leading to autistic-like phenotypes in mice. Proc Natl Acad Sci USA 109 (52) 21516–21521. 23236174 10.1073/pnas.1216988110 PMC3535652

