# Supplementary figures and images for: Mapping Pathological Phenotypes in a Mouse Model of CDKL5 Disorder
Source: PLoS One. 2014 May 16;9(5):e91613. doi: 10.1371/journal.pone.0091613 (PMC4023934; doi:10.1371/journal.pone.0091613)

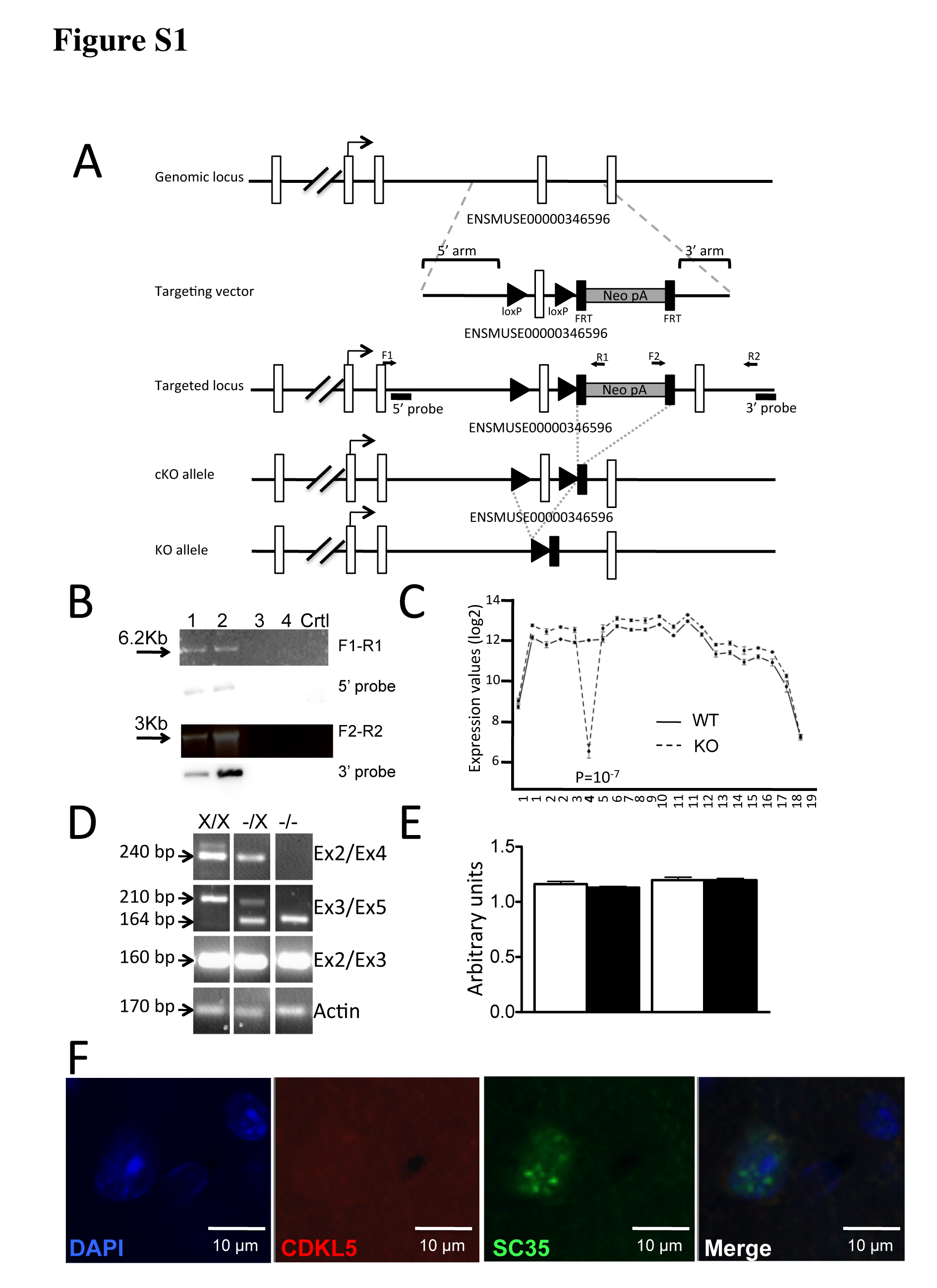

Supplement: Figure S1 — Generation and validation of Cdkl5 knockout mice. (A) Genomic organization of the Cdkl5 locus showing critical exon 4 (ENSMUSE00000346596), the targeting construct, successfully targeted Cdkl5 locus (genotyping primers indicated by arrows), FRT-deleted conditional Cdkl5 knockout allele, and Cre-deleted constitutive Cdkl5 knockout allele used in the present study. (B) Confirmation of homologous recombinants by long-range PCR at 5′end (top panel) and 3′end (bottom panel). PCR products of positive clones (lane 1 and 2) showed specific signals when hybridized with 5′ and 3′ radioactive-labelled DNA probes. No signal was detected in PCR-negative samples, consistent with them being non-homologous or unmodified (lane 3 and 4). (C) mRNA expression of Cdkl5 exons as estimated by Affymetrix microarray hybridization in wild-type and Cdkl5 knockout brain confirmed absence of the deleted exon 4 in knockout mice, but normal expression of remaining exons consistent with an escape from nonsense-mediated decay. (D) Semi-quantitative PCR on total brain RNA from female wild-type and Cdkl5 mutant mice with primers spanning exons confirmed an absence of exon 4, but normal levels of exons 2, 3, and 5 in mutant mice. (E) Quantitative real-time PCR on total brain RNA from female wild-type and Cdkl5 knockout mice revealed normal levels of expression of upstream (exon 2–3) and downstream (exon 9–10) Cdkl5 exons. (F) Anti-Cdkl5 and SC35 immunofluorescence analysis of S1 cortex brain sections from adult male knockout (KO) mice. (Scale bar 10 µm). (TIF) [file pone.0091613.s001.tif]

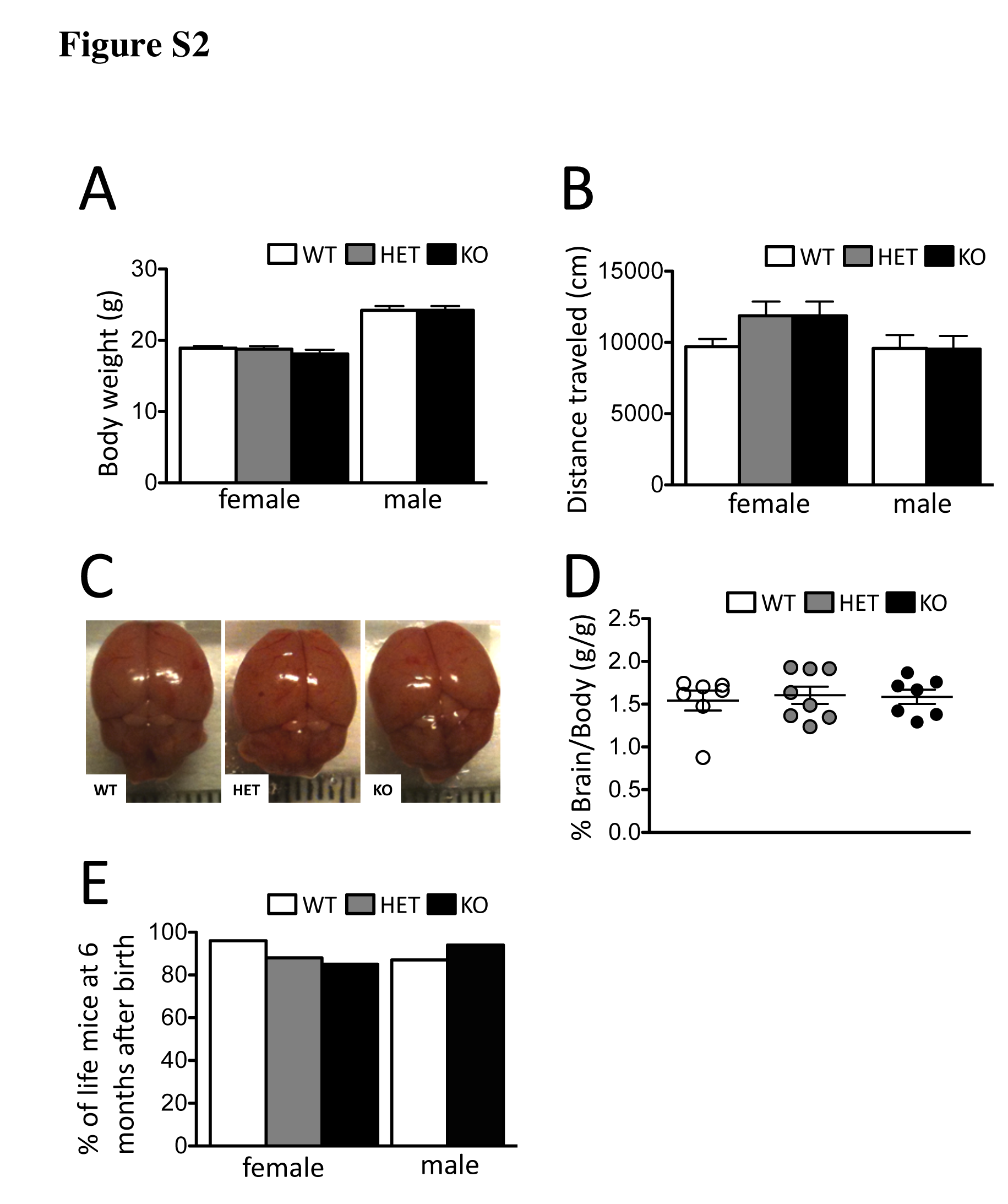

Supplement: Figure S2 — Normal body and brain weight and novelty-induced locomotion in Cdkl5 knockout mice. (A) No difference was observed in body weight of female and male wild-type and Cdkl5 mutant mice (6 weeks old). (B) No difference was detected in total distance travelled in a novel open arena by adult female and male wild-type and Cdkl5 mutant mice (female: WT, N = 10, HET, N = 9, KO, N = 10; male: WT, N = 10, KO, N = 11). (C) Representative images of dissected brains from female wild-type, heterozygous, and homozygous Cdkl5 knockout mice. (D) No difference in relative brain to body weight was detected between genotypes (WT, N = 6, HET, N = 8, KO, N = 7; mean ± SEM). (E) Normal viability was observed of female and male wild-type and Cdkl5 mutant mice (at 6 months of life) (female: WT, N = 48/50, HET, N = 24/27, KO, N = 17/20; male: WT, N = 34/39, KO, N = 51/54, mean ± SEM). (TIF) [file pone.0091613.s002.tif]

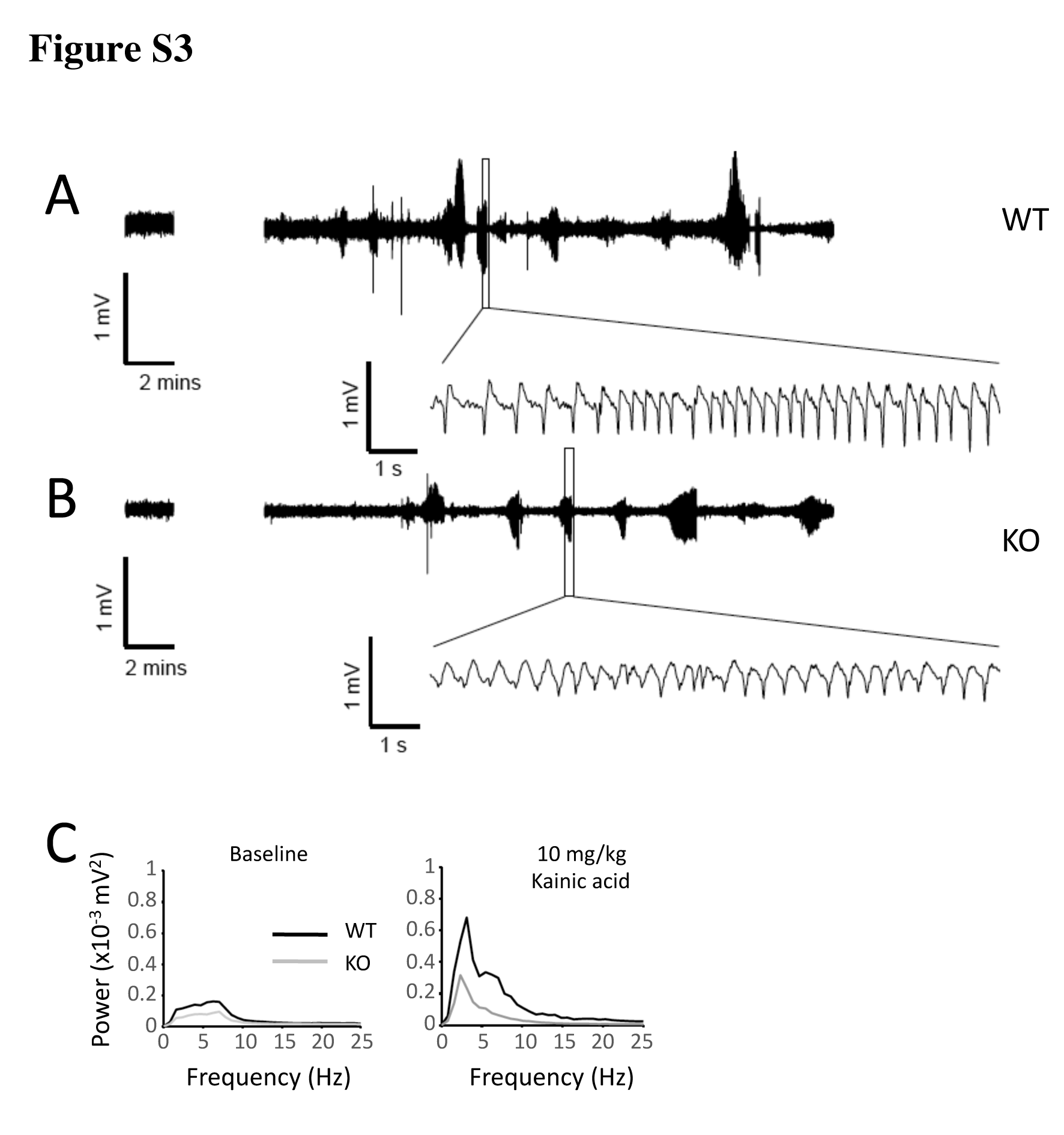

Supplement: Figure S3 — Seizure response in Cdkl5 knockout mice backcrossed to DBA genetic background. Representative electroencephalogram (EEG) traces recorded from surface electrodes placed over the somatosensory cortex in freely moving male (A) wild-type (WT) and (B) Cdkl5 knockout (KO) mice following 3 generations backcrossing to the DBA2/J strain. Power spectra of EEG recordings showed decreased power in Cdkl5 knockouts when compared to wild-type littermates at low frequencies both under (C) baseline conditions and (D) in mice injected with kainic acid (25 mg/kg, i.p.; mean ± SEM; WT: N = 3, KO: N = 3). (TIF) [file pone.0091613.s003.tif]

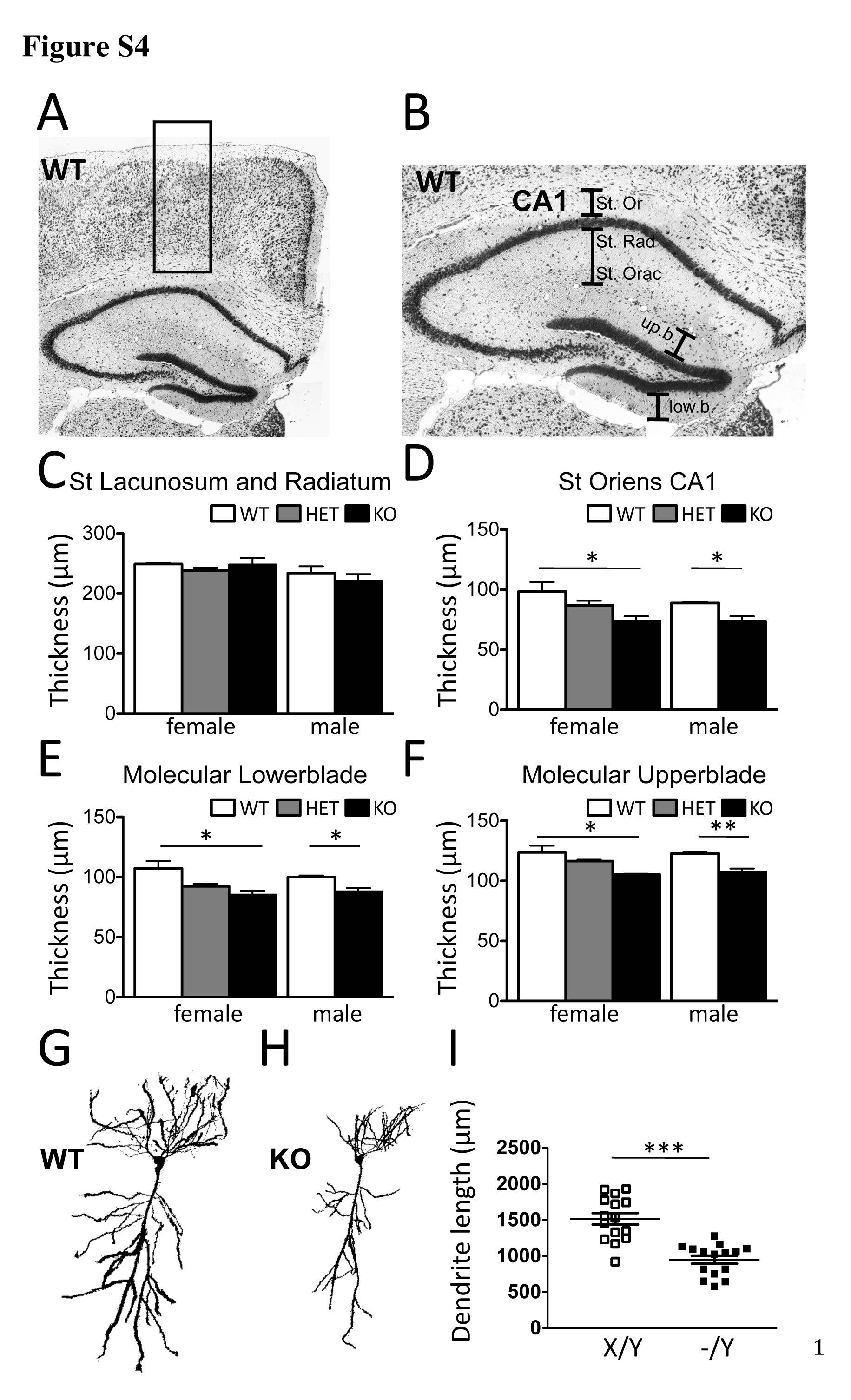

Supplement: Figure S4 — Reduced thickness of cortical and hippocampal layers in Cdkl5 knockout mice. (A) Representative image showing region of S1 cortex used for quantification of cortical thickness in wild-type and Cdkl5 mutant mice. (B) Representative image showing features used for quantification of hippocampal layer thickness in wild-type and Cdkl5 mutant mice. (C–F) A significant decrease in thickness was observed in hippocampal CA1 stratum oriens (but not stratum laconosum or radiatum) and the lower and upper blades of dentate gyrus molecular layer in female and male Cdkl5 mutant mice compared to wild-type littermates (female: WT, N = 3, HET, N = 3, KO, N = 3; male: WT, N = 3, KO, N = 3; N = 9–13 sections for each genotype) (mean ± SEM; *P<0.05, **P<0.01). (G,H) Representative images of reconstructed neurons from adult male wild-type (WT, G) and Cdkl5 knockout (KO, H) mice. (I) Total dendrite length was significantly reduced in male Cdkl5 knockout mice (X/Y, N = 15; -/Y, N = 15, mean ± SEM, *P<0.05, **P<0.01, ***P<0.001). (TIF) [file pone.0091613.s004.tif]

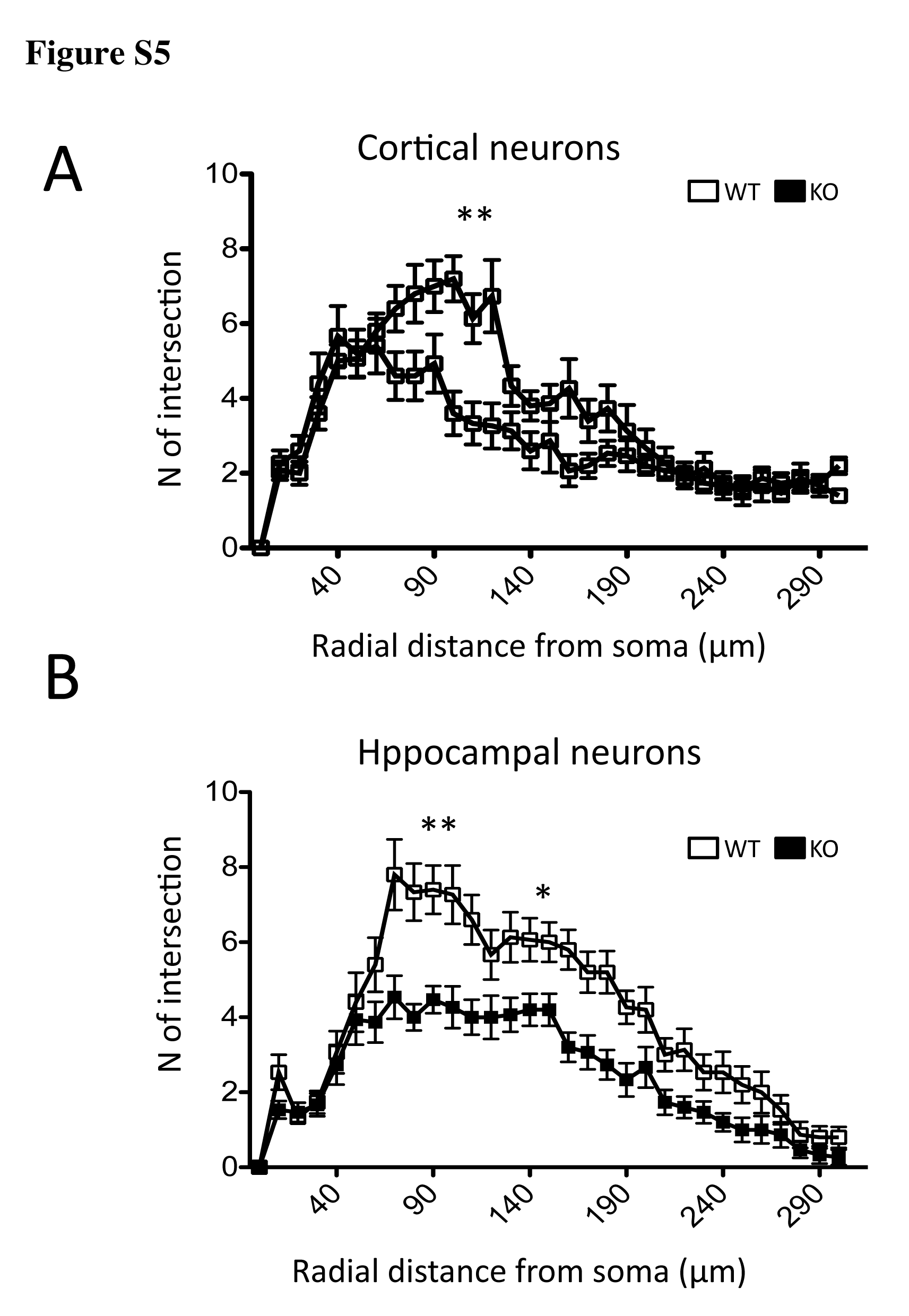

Supplement: Figure S5 — Reduced dendrite complexity in Cdkl5 knockout mice. Classical Sholl analysis with radii increasing in 20 µm increments. Sholl analysis measures apical dendrite length within each sphere plotted against radius from soma. Significant decreases in the numbers of intersections between the dendrites and the Sholl circles from the neuronal somata occurred only between 100 µm and 130 µm in cortical neurons (A) and between 80 µm and 120 µm and 140 µm and 160 µm in hyppocampal dendrite (B) in mutant Cdkl5 male mice (Figure S5; P<0.05, Tukey test). (TIF) [file pone.0091613.s005.tif]

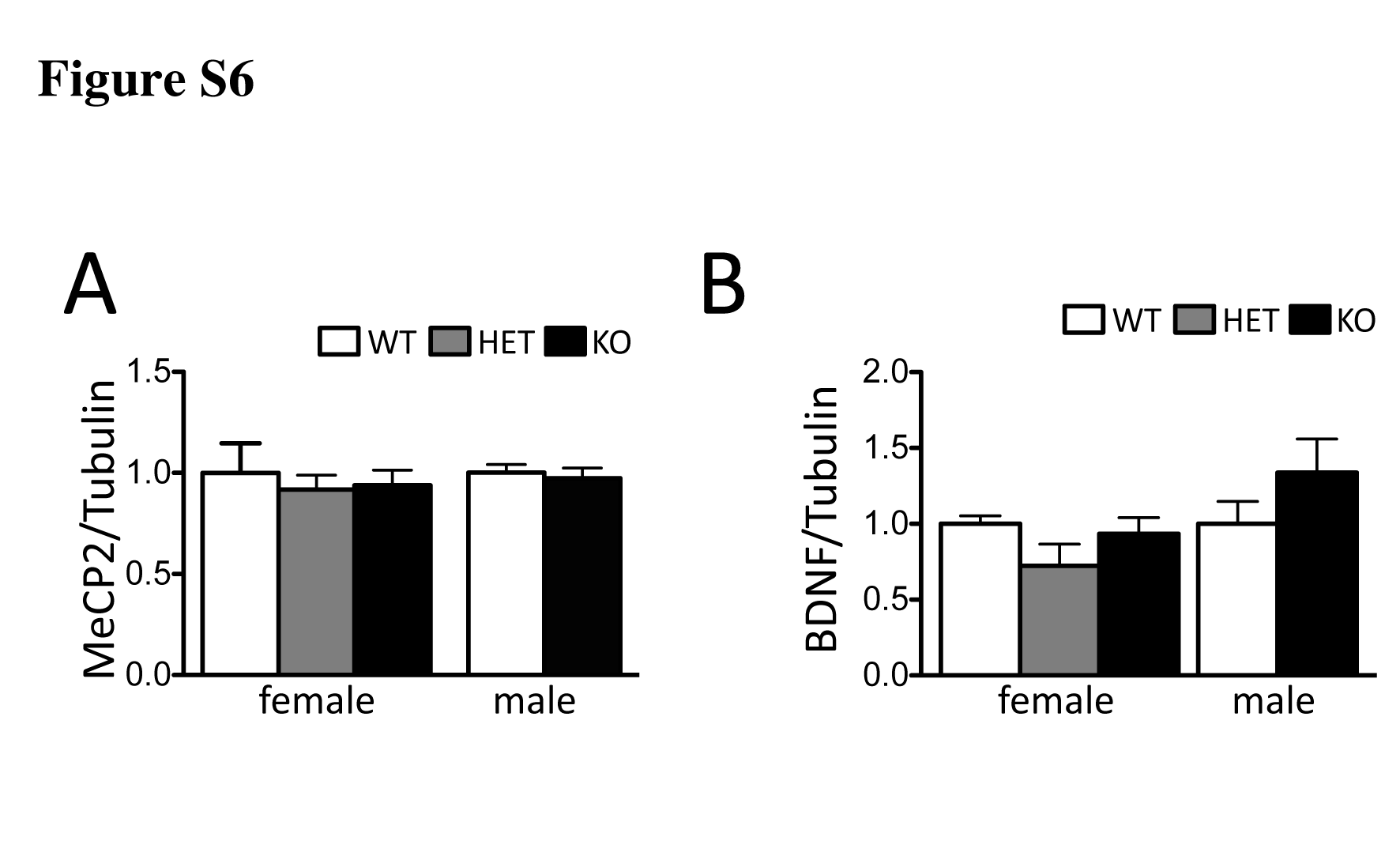

Supplement: Figure S6 — No change observed in Mecp2 and BDNF levels in Cdkl5 knockout mice. Quantification of western blot data failed to detect a change in immunostaining against (A) Mecp2, and (B) BDNF protein in mutant Cdkl5 mice compared with wild-type controls (female: WT, N = 10, HET, N = 6, KO, N = 10; male: WT, N = 5, KO, N = 6; mean ± SEM) (mean ± SEM; *P<0.05, **P<0.01). (TIF) [file pone.0091613.s006.tif]

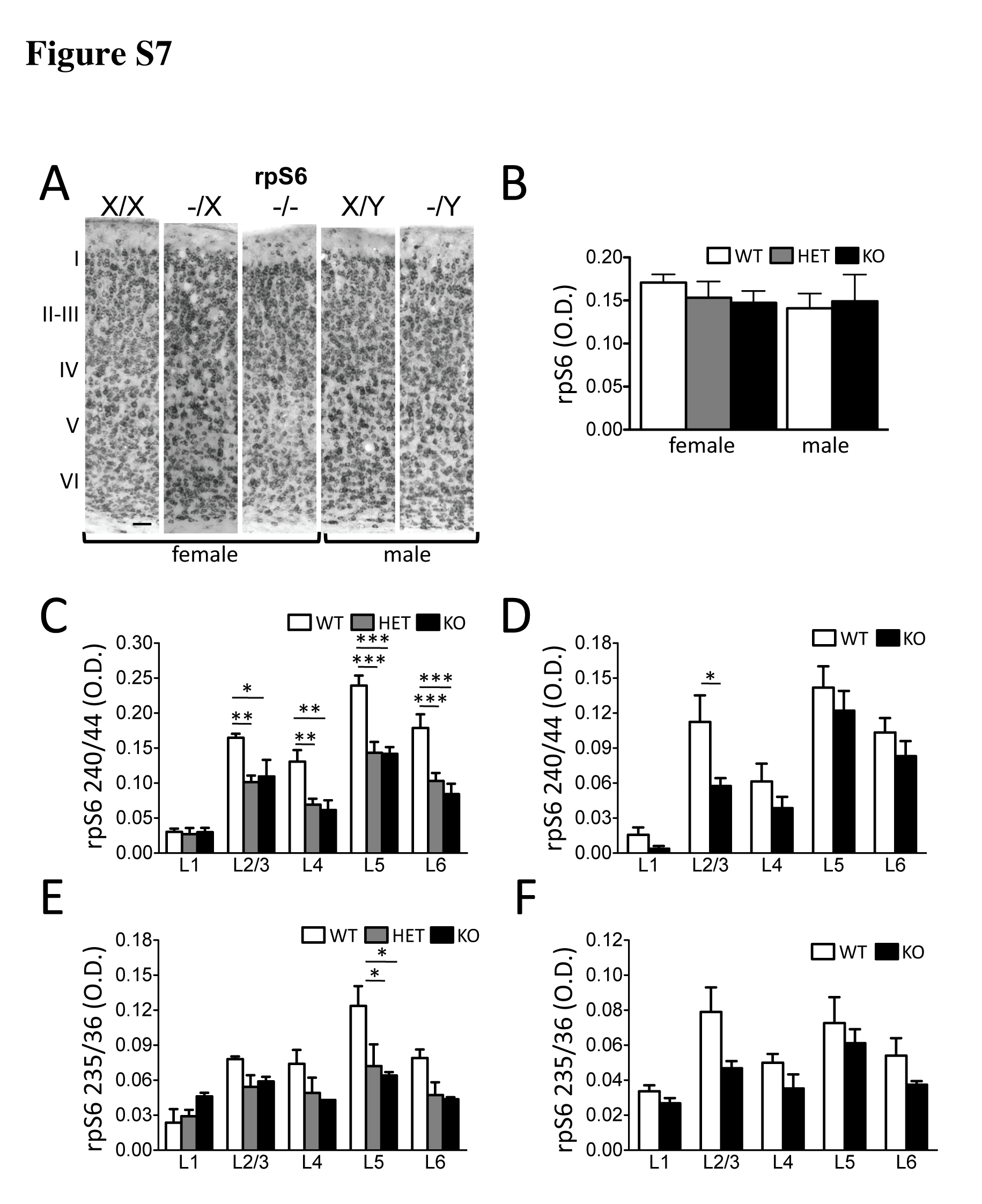

Supplement: Figure S7 — Reduced p-rpS6 protein in Cdkl5 knockout mice. Representative micrographs showing the immunohistochemistry for total rpS6 (A) in the S1 cortex of both female and male wild-type and Cdkl5 mutant mice. Quantitation of immunoreactivity signals revealed no change in total rpS6 protein (B) in both male and female mutants, a significant decrease of phospho-rpS6 (240/244) in (C) female and (D) male mutants, and a decrease of phospho-rpS6(235/236) that was only significant in layer V of (E) female mutants, while it shows only a trend in (F) male KOs (female: WT, N = 4, HET, N = 6, KO, N = 3; male: WT, N = 4, KO, N = 4; mean ± SEM; *P<0.05, **P<0.01, ***P<0.001). (TIF) [file pone.0091613.s007.tif]

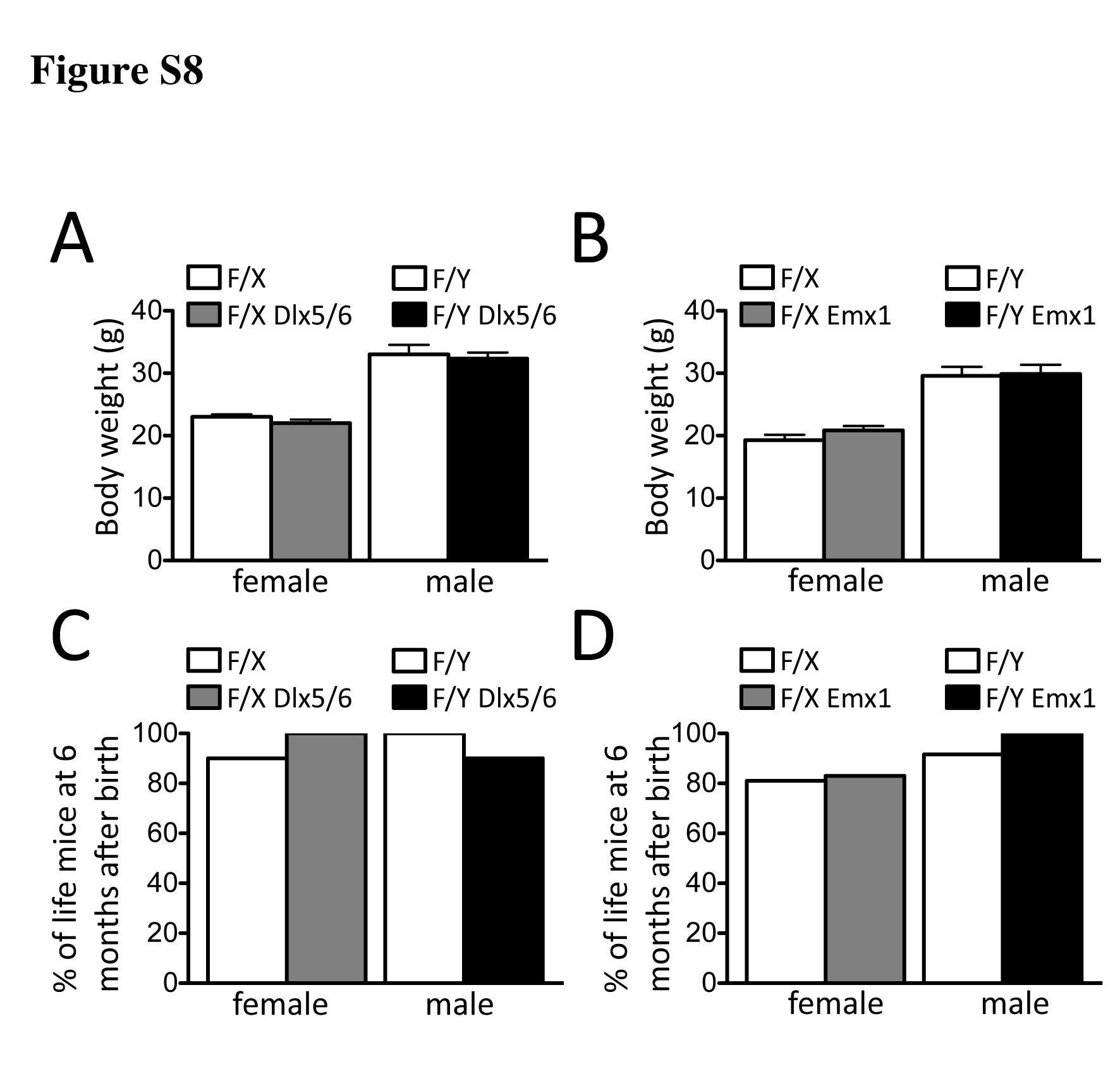

Supplement: Figure S8 — Normal body and brain weight (A) No difference was observed in body weight of heterozygous female and hemizygous male (A) Dlx5/6- (F/X, N = 8; F/X-Dlx5/6::Cre, N = 7; F/Y, N = 5; F/Y-Dlx5/6::Cre, N = 11) and (B) Emx1 (F/X, N = 12; F/X-Emx1::Cre, N = 8; F/Y, N = 12; F/Y-Emx1::Cre, N = 10) conditional Cdkl5 knockout mice at 8 weeks of life. Normal viability was observed at 6 months of life of heterozygous female and hemizygous male (C) Dlx5/6- (F/X, N = 9/10; F/X-Dlx5/6::Cre, N = 6/6; F/Y, N = 5/5; F/Y-Dlx5/6::Cre, N = 9/10) and (D) Emx1 (F/X, N = 9/11; F/X-Emx1::Cre, N = 5/6; F/Y, N = 11/12; F/Y-Emx1::Cre, N = 9/9) conditional Cdkl5 knockout. (TIF) [file pone.0091613.s008.tif]
